# Fluidics system for resolving concentration-dependent effects of dissolved gases on tissue metabolism

**DOI:** 10.7554/eLife.66716

**Published:** 2021-11-04

**Authors:** Varun Kamat, Brian M Robbings, Seung-Ryoung Jung, John Kelly, James B Hurley, Kenneth P Bube, Ian R Sweet

**Affiliations:** 1 University of Washington Medicine Diabetes Institute, University of Washington Seattle United States; 2 Department of Biochemistry, University of Washington Seattle United States; 3 VICI Metronics Seattle United States; 4 Department of Mathematics, University of Washington Seattle United States; University of California, Berkeley United States; The University of Texas at Austin United States

**Keywords:** hydrogen sulfide, oxygen, hypoxia, insulin secretion, calcium, fluidics, Mouse, Rat

## Abstract

Oxygen (O_2_) and other dissolved gases such as the gasotransmitters H_2_S, CO, and NO affect cell metabolism and function. To evaluate effects of dissolved gases on processes in tissue, we developed a fluidics system that controls dissolved gases while simultaneously measuring parameters of electron transport, metabolism, and secretory function. We use pancreatic islets, retina, and liver from rodents to highlight its ability to assess effects of O_2_ and H_2_S. Protocols aimed at emulating hypoxia–reperfusion conditions resolved a previously unrecognized transient spike in O_2_ consumption rate (OCR) following replenishment of O_2_, and tissue-specific recovery of OCR following hypoxia. The system revealed both inhibitory and stimulatory effects of H_2_S on insulin secretion rate from isolated islets. The unique ability of this new system to quantify metabolic state and cell function in response to precise changes in dissolved gases provides a powerful platform for cell physiologists to study a wide range of disease states.

## Introduction

### A critical need for instrumentation to study the effect of dissolved gases

Oxygen (O_2_) is a fundamental determinant of cell survival and function in mammalian tissues. In most cells, the majority of ATP is generated by oxidative phosphorylation, driven by a series of redox reactions in which O_2_ is the ultimate electron acceptor. Hypoxia is linked to many diseases including stroke, cancer, and diabetic complications. In addition to O_2_, trace gases produced by cells (H_2_S, NO, and CO) act as signals to regulate cellular and mitochondrial function (Prabhakar and Semenza, 2012). Ischemia–reperfusion injury is a condition common to many disease states and it is thought that during reoxygenation a burst of reactive O_2_ species (ROS) occurs that can damage proteins, lipids, and nucleic acids (Chouchani et al., 2016; Zweier et al., 1987). Yet despite the scientific and clinical importance of dissolved gases, quantitative methods to measure the real-time effects of dissolved gases on intact tissue are not available. Investigators who have studied trace gases and who are characterizing drugs to attain the same benefits (Chen et al., 2020) almost exclusively use aqueous based surrogates/donors of gas. The equivalence of these drugs to the gases they are supposed to mimic has not been tested (Chen et al., 2020). Some investigators have bubbled gas directly into media, but this precludes adding essential protein to the media due to foaming. In addition, the study of dissolved gases is hampered by the volatility of dissolved gases under conditions where the headspace is not supplied with equilibrium levels of the gas (DeLeon et al., 2012). Thus, there is a strong need to develop technology that enables the study of both abundant and trace dissolved gases. The system we describe here does these analyses both quantitatively and reproducibly.

### A flow culture/assessment system that exposes tissues to precise levels and durations of dissolved gases

We developed a flow culture system according to three fundamental and essential specifications needed to assess effect of gases on tissue: (1) maintain tissue function and viability under continuous flow culture conditions; (2) continuously monitor parameters that reflect intracellular changes in metabolism in real time; (3) precisely control the aqueous and gas-phase composition of the media bathing the tissue. Although there are many systems readily available that have some components needed to investigate effects of dissolved gas, none incorporate all three. Commercially available hypoxia chambers (for instance Baker Ruskinn Cell Culture Workstations) control steady-state levels of dissolved gas and have been effectively and most commonly used for hypoxia studies (Jaakkola et al., 2001; Epstein et al., 2001). Microfluidics systems have been developed for establishing cell and tissue models where the three-dimensional structure and cell-to-cell interactions of native tissue can be recreated, which can be used under steady-state gas compositions (Ren et al., 2013; Manafi et al., 2021; Kimura et al., 2018). In addition, some investigators have used the Oroboros machine to characterize the effects of O_2_ levels on metabolic processes (Stepanova et al., 2019). However, none of these methods are designed for implementing, and assessing real-time effects of, rapid changes in dissolved gas concentrations. The Seahorse flux analyzer measures OCR and extracellular acidification rate (mostly from glycolysis and the TCA cycle) on cell monolayers (Wu et al., 2007) and has been extensively utilized across many fields. However, it is not designed to maintain tissue in physiological buffers or to control dissolved gas levels.

In previous reports, we described an earlier version of our flow culture system and demonstrated its ability to maintain a range of tissues (including islets, retina, liver, and brain) over hours and days (Neal et al., 2016; Neal et al., 2015; Sweet et al., 2009; Gilbert et al., 2008; Weydt et al., 2006; Bisbach et al., 2020) while continuously assessing metabolic and functional effects of test compounds. This report highlights the incorporation of technology to precisely control both abundant gases (such as O_2_, CO_2_, and N_2_), by using a countercurrent flow device that promotes equilibration between inflow media and premixed gas, and also trace gases, by novel application of permeation tubes. Permeation tubes are commonly used devices for calibration of safety equipment that detect toxic gases such as CO and H_2_S. They consist of liquified gas housed under pressure in a metal jacket, where the gas continuously leaks through a membrane at a steady and accurately calibrated rate. We present in detail the components and operation of the system in the Methods section, and then illustrate the utility of the system to measure the effects of hypoxia followed by reoxygenation on two tissues, pancreatic islets and retina. We also demonstrate how this instrument can be used to quantify the effects of H_2_S on islet function and liver energetics.

### Assessment of O_2_-sensitive processes: OCR, reductive state of cytochromes, and rate of lactate and pyruvate production

**Figure 1.**
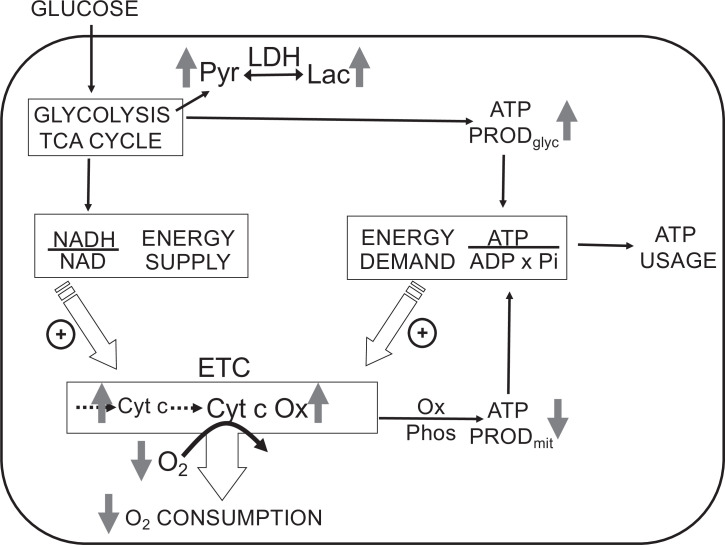
Direct control of O_2_ consumption rate (OCR): energy supply vs. energy demand vs. O_2_. *Schematic depicting three mechanisms mediating OCR*. (1) The supply of reduced electrons generated by metabolism of fuel. (2) The usage of ATP by energy-utilizing cellular function yielding ADP (a major regulator of OCR). (3) The concentration of dissolved O_2_. The concomitant measurement of reduced cytochromes and O_2_ allows for the distinction between the three mechanisms mediating observed changes in OCR. The vertical arrows depict the changes that are acutely affected by changes in O_2_. Low O_2_ leads to increased reductive state of cytochrome c and cytochrome c oxidase, decreased OCR. In some tissue types, the decrease in ATP production by oxidative phosphorylation is compensated by increased ATP generation from glycolysis (the Pasteur effect).

The most direct endpoints with which to assess the acute effects of O_2_ are components of the electron transport chain (ETC), first and foremost OCR. However, measurements of OCR alone cannot identify the mechanisms that are mediating changes in OCR. Under physiological conditions, OCR can increase in response to changes in substrate supply (increased supply of electrons generated from metabolism) and/or demand (as stimulated by ADP Chance and Williams, 1956b; Wilson et al., 1979; Figure 1). Metabolism of fuels is reflected by a proportional change in cytochrome reduction and OCR: as the number of electrons bound to cytochromes increase, OCR increases by mass action (a ‘push’ system). In contrast, increased ATP usage by energy-utilizing cell functions (importantly ion flux and biosynthesis) with a corresponding increase in ADP leads to increased OCR, but without increased cytochrome c reduction (a ‘pull’ system) (Brown, 1992; Sweet and Gilbert, 2006). In this way, OCR changes mediated by substrate supply vs. ATP usage can be distinguished, and these fingerprints are informative in understanding mechanisms mediating changes in the ETC induced by H_2_S and O_2_.

In addition to regulating ETC activity, O_2_ can also influence glycolysis (known as the Pasteur effect Krebs, 1972; Figure 1). Lactate and pyruvate accumulation and release are determined by relative rates of glycolysis, lactate dehydrogenase (LDH), pyruvate dehydrogenase, mitochondrial and plasma membrane transporters, and the redox state of the cytosol. Due to the equilibrium status of LDH, [lactate]/[pyruvate] ratio is proportional to the cytosolic redox state ([NADH]/[NAD]) (Williamson et al., 1967), so by measuring both lactate and pyruvate, changes in the rate of lactate production rate due to alterations in glycolytic flux vs. cytosolic redox state can be distinguished. The concomitant measurement of O_2_, OCR, cytochrome c, cytochrome c oxidase, lactate, and pyruvate production provides a comprehensive dataset to assess the multitude of biochemical and functional effects of O_2_ and other gases.

### Assessment of H_2_S effects on insulin secretion rate (ISR) from isolated pancreatic islets

Like O_2_, H_2_S also interacts directly with the ETC, where it can be both stimulatory and inhibitory. H_2_S can inhibit cytochrome c oxidase (Khan et al., 1990) and it also can donate electrons to cytochrome c (Vitvitsky et al., 2018). However, with respect to pancreatic islets, all previous reports have described only inhibition of ISR (Ali et al., 2007; Niki and Kaneko, 2006; Wu et al., 2009; Yang et al., 2005; Bełtowski et al., 2018; Tang et al., 2013). Based on the ability of H_2_S to both increase and decrease ETC activity, we chose to demonstrate the technical caliber of our system by testing the hypothesis that H_2_S would both stimulate and inhibit ISR depending on its concentration. Flow culture systems are well suited to measure changes in ISR in response to changes in perifusate composition (Lacy et al., 1972). Secretogogs that affect ISR by isolated islets including glucose, arginine, amino and fatty acids, acetylcholine, GLP-1 as well as sulfonylureas and GLP-1 analogs, also manifest their effects in vivo. Therefore, isolated islets are a validated and highly relevant model with which to test our flow culture system. To evaluate the commonly asserted assumption that donors of H_2_S yield the same effects as direct exposure to H_2_S, we also compared the effects of NaHS, a commonly used donor of H_2_S to direct exposure to dissolved H_2_S.

## Results

### Measurement of OCR, reduced cytochrome c, and ISR by pancreatic islets in the face of changing inflow O_2_

**Figure 2.**
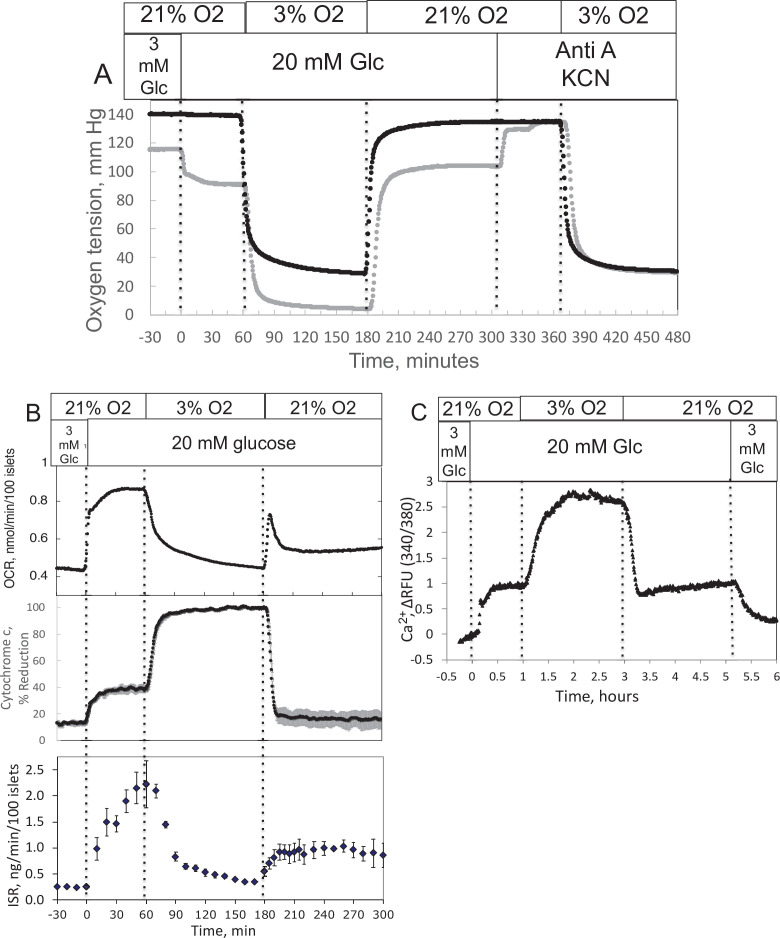
Effect of glucose and low O_2_ on transient and steady-state electron transport chain (ETC), and insulin secretion rate (ISR) and intracellular Ca^2+^ in pancreatic islets. Islets (900/channel) were handpicked with a P200 pipet and after mixing with Cytodex beads (1 μl/10 islets) loaded into the perifusion chamber, and the flow rate was set to 150 μl/min with Krebs–Ringer bicarbonate buffer containing 3 mM glucose for 90 min. At time = 0, glucose concentration was raised to 20 mM for 45 min; subsequently, O_2_ was decreased to 3% for 2 hr, and then returned to 21%. (**A**) The protocol generated inflow and outflow O_2_ profiles such as shown. Following the completion of the protocol, 12 μg/ml antimycin A (aA) was added for 25 min, and then 3 mM potassium cyanide (KCN), and the hypoxia protocol was repeated while islet respiration was suppressed in order to characterize delay and dispersion due to the separation in space of inflow and outflow sensors. (**B**) Calculated values of O_2_ consumption rate (OCR) representative data from an *n* of 3 (average recovery = 0.55 ± 0.07), reduced cytochrome c (*n* = 2), and ISR (*n* = 2) were plotted as described in the Methods section. (**C**) In a separate illustrative experiment, intracellular Ca^2+^ in islets was imaged and quantified using the same protocol except glucose was also decreased back to 3 mM glucose at the end of the experiment. Raw data can be found in a Source Data file named ’Figure 2—source data 1’. Figure 2—source data 1.Effect of glucose and oxygen on islet function, metabolism and signaling.

Ischemia–reperfusion is a stress to tissues that occurs under a range of pathophysiologic conditions, and it is recognized that damage from hypoxia can occur both from the period of decreased energy production and at the time when O_2_ is replenished. Accordingly, we evaluated the ability of our system to measure the recovery of metabolism and function following a period of decreased O_2_ levels. Isolated rat islets were placed into the perifusion chamber and perifused for 90 min with Krebs–Ringer bicarbonate buffer containing 3 mM glucose and equilibrated with 21% O_2_/5% CO_2_/balance N_2_. Changes in OCR, cytochrome c reduction state, and ISR were measured in response to increased glucose (20 mM), decreased O_2_ (by switching to a gas tank supplying the gas equilibration system that contained 3% for 2 hr), and the return of O_2_ to 21% (Figure 2). To measure OCR, both the inflow and outflow O_2_ concentrations were measured (Figure 2A), and the data were then processed by convolution techniques described in the Methods section. After transformation of the inflow O_2_ using Equation 4 and the transfer function generated with data obtained in the presence of potassium cyanide (KCN) to account for the delay and dispersion of the perifusion chamber, OCR was calculated from Equation 5. As expected, OCR increased with increased glucose concentration, and decreased with lower O_2_ tension (Figure 2B). Notably, there was a transient spike of OCR when O_2_ in the media was restored. OCR then approached a steady state that was about 55% of the prehypoxic level of OCR. One limitation of the system arises when defining the O_2_ levels that tissue is exposed to, when in fact there is a gradient from the inflow to the outflow. This uncertainty can be minimized by increasing flow rate thereby decreasing the difference between inflow and outflow concentrations; however, as the difference gets smaller, the resolution of the method decreases.

The reduced state of cytochrome c was concomitantly measured with OCR (Figure 2B). Previous reports described an equilibrium with respect to the flow of electrons between NADH and cytochrome c (Wilson et al., 1974a; Wilson et al., 1974b), and their reductive state represents a balance between the supply of electrons generated by metabolism of fuels and use of electrons to drive proton translocation and ATP production. Consistent with these scenarios, glucose provided more reducing power to drive cytochrome c to its reduced state (in parallel with OCR), whereas hypoxia favored the reduced state of cytochrome c by slowing its oxidation. Following the return to 21% O_2_, cytochrome c reduction reached a steady state of 42% of the prehypoxic levels, consistent with incomplete recovery of OCR. A particularly powerful feature of the systems approach is realized when tissue function can be measured concomitantly with measures of ETC. Fractions were collected during the protocol that were later assayed for insulin (Figure 2B). Stimulation of ISR by glucose in the presence of 21% O_2_ was suppressed in low O_2_, and ISR recovered to 44% of its original level of stimulation after reoxygenation.

### Measurement of calcium (Ca^2+^) in response to decreased O_2_ by islets

As fluorescence imaging is a powerful and widely used modality to assess many intracellular signals including but not limited to Ca^2+^ (Rountree et al., 2014), NADH (Bennett et al., 1996; Pedersen et al., 2013), mitochondrial membrane potential (Pertusa et al., 2002), ATP (Hodson et al., 2014), and ROS (Neal et al., 2016) in islets, we demonstrated the control of dissolved gas for this modality. We measured the effect of glucose and hypoxia on islet intracellular Ca^2+^ with a protocol similar to the one we used for OCR except that glucose was lowered back to 3 mM at the end of the experiment (Figure 2C). As expected, intraislet Ca^2+^ increased in response to the increase in glucose. Subsequently, in response to a decrease in O_2_, Ca^2+^ fluorescence rose 3-fold, presumably reflecting a loss of energy-dependent pumping of Ca^2+^ out of the cells. In contrast to OCR and ISR parameters, Ca^2+^ recovered fully to prehypoxic levels when O_2_ was returned to normal levels.

### Measurement of lactate/pyruvate production and release by perifused INS-1 832/13 cells in response to changes in O_2_

**Figure 3.**
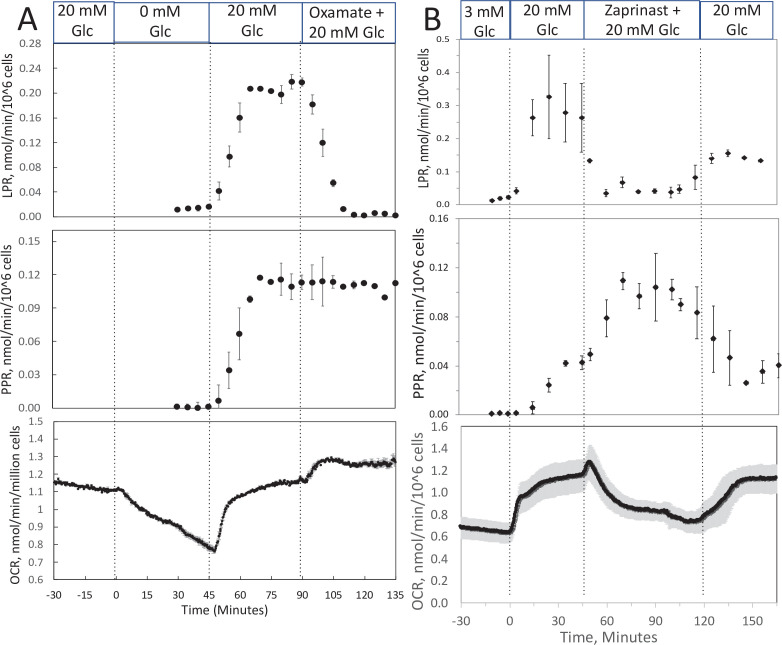
Effect of glucose and inhibitors of lactate dehydrogenase (LDH) and mitochondrial pyruvate carrier (MPC) on O_2_ consumption rate (OCR), lactate, and pyruvate production rate by INS-1 832/13 cells. (A) The effects of glucose and then oxamate (50 mM, an inhibitor of LDH) on OCR, lactate production rate, and pyruvate production rate at the times indicated in the figure were measured. (**B**) The effects of glucose and then zaprinast (200 μM, an inhibitor of MPC) on OCR, lactate production rate, and pyruvate production rate. Data for both plots are the average ± standard error (SE), *n* = 2. Raw data can be found in a Source Data file named ‘Figure 3—source data 1’. Figure 3—source data 1.Effect of low oxygen on metabolism in isolated retina.

To track shifts between glycolytic and mitochondrial energy generation in real time, fractions collected from the outflow were assayed for lactate and pyruvate. We predicted that extracellular ratios of these two analytes reflect intracellular regulation of these two compounds. To evaluate this, we measured the response to inhibitors of LDH and mitochondrial transport of pyruvate in INS-1 832/13 cells (henceforth referred to as INS-1 cells). INS-1 cells were used instead of islets because most of the pyruvate made in islets is transported into mitochondria (MacDonald, 1990) since they do not have significant capacity for plasma membrane transport of lactate or pyruvate (Ishihara and Wollheim, 2000). Oxamate, an inhibitor of LDH, rapidly and completely suppressed lactate release from cells (Figure 3A), showing the tight relation between production of lactate from LDH and appearance of lactate in the outflow. Glucose-stimulated OCR obtained with INS-1 cells was similar to the values obtained previously (Jesinkey et al., 2019) and about half of what was measured using a Seahorse with metabolite-rich media (Dover et al., 2018). The rate of INS-1 OCR is about 1/4th of islet OCR on a per cell basis, probably reflecting loss of lactate in INS-1 cells that does not occur in islets, in addition to lower rates of synthesis and secretion of insulin. Somewhat surprisingly, pyruvate did not increase. However, OCR increased suggesting that the decrease in flux from pyruvate to lactate was counterbalanced by an increase in flux of pyruvate into the mitochondria. Blocking transport of pyruvate into mitochondria with [zaprinast a blocker of the mitochondrial pyruvate carrier (MPC; Du et al., 2013)] led to a rapid increase in pyruvate release from cells (Figure 3B) and diminished OCR. Why lactate production decreased is not clear from the data but could be explained by a decrease in the cytosolic redox state (NADH/NAD) by the increased activity of the malate/aspartate shuttle. Thus, the rapidity of the changes in lactate and pyruvate production in response to changes in LDH and MPC indicates that membrane transport is fast, and at least in this cell line, extracellular lactate and pyruvate will reflect changes in intracellular events controlling lactate and pyruvate with a time delay of no more than a few minutes. To ensure that this is the case, these experiments would have to be done in whatever tissue is being investigated.

### Measurement of OCR, cytochrome c, lactate, and pyruvate by perifused retina before and after a period of hypoxia

**Figure 4.**
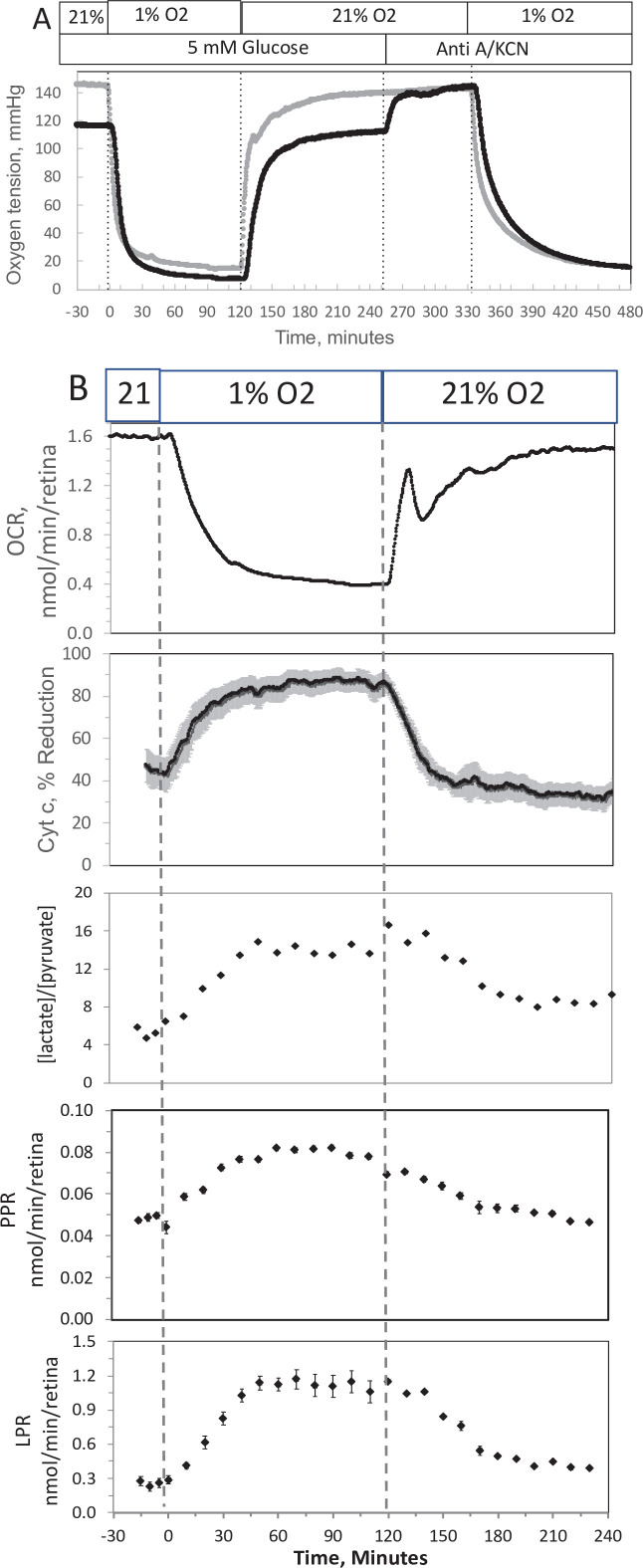
Effect of low O_2_ on transient electron transport chain (ETC), and lactate/pyruvate in isolated retina. (A) Four retinas (16 pieces) per chamber were loaded into the flow chamber, where each group of 4 retinal pieces were separated by a 3 μl layer of Cytodex beads. The tissue was sandwiched on the top and bottom by 50 μl of Cytopore beads and both layers were held in place with a porous frit (Interstate Specialty Products, Suton, MA, Cat no. POR 4894, cut to 4.2 mm diameter and 0.25 in long). Ninety minutes after loading the retina into the system (flow rate = 130 μl/min), the O_2_ tank was switched one containing 1% O_2_ for 2 hr, and subsequently returned to 21%. (**A**) The protocol generated inflow and outflow O_2_ profiles such as shown. Following the completion of the protocol, 12 μg/ml antimycin A (Anti A) was added for 20 min, and then 3 mM potassium cyanide (KCN), and the hypoxia protocol was repeated while retinal respiration was suppressed in order to characterize delay and dispersion due to the separation in space of inflow and outflow sensors. (**B**) Measurements of OCR representative data from an *n* of 6 (average recovery = 0.83 ± 0.03), reduced cytochrome c (*n* = 6), lactate and pyruvate production rates (*n* = 2), and [lactate]/[pyruvate] are shown. Raw data can be found in a Source Data file named ‘Figure 4—source data 1’. Figure 4—source data 1.Effect of low oxygen on metabolism in isolated retina.

**Figure 5.**
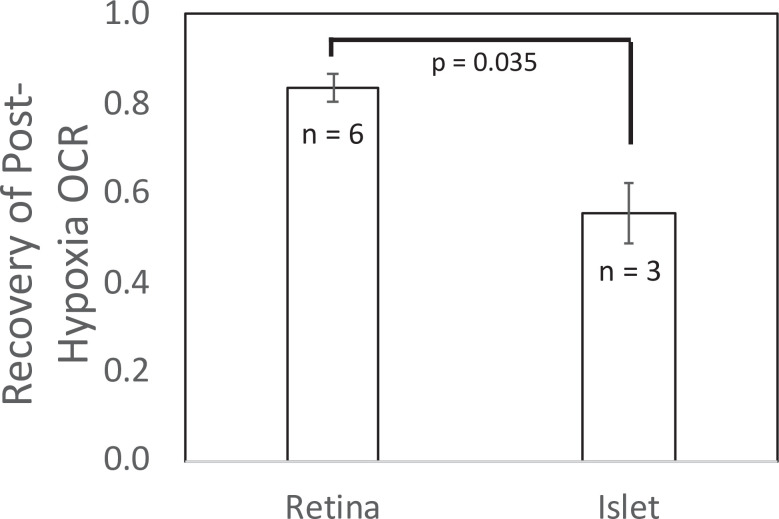
Recovery of O_2_ consumption rate (OCR) after hypoxia relative to OCR prehypoxia values in retina and islets. Recovery data from experiments shown in Figures 2 and 4. *t*-Test result: p = 0.035, for retina, *n* = 6, and for islets, *n* = 3. Raw data can be found in a Source Data file named ‘Figure 5—source data 1’. Figure 5—source data 1.Recovery of oxygen consumption after hypoxia.

In order to compare results with islets to those obtained by a tissue that is less sensitive to hypoxia, experiments were carried out on isolated retina, a tissue that normally resides at low O_2_ (Bisbach et al., 2020). Similar to the measurement in islets, the inflow and outflow O_2_ were measured (Figure 4A), and OCR was calculated after convolution of the inflow data. OCR decreased at low O_2_, and then manifested a transient spike in response to reoxygenation (Figure 4B). However, in contrast to islets, OCR by retina then approached a much higher recovery (a steady state of 83% of the prehypoxic rate). Reduced cytochrome c increased to maximal levels during 1% O_2_ and stayed at this level throughout the 2 hr of low O_2_ (Figure 4B). Consistent with OCR data, upon return of O_2_ to 21% cytochrome c reduction recovered to 79% of prehypoxic levels. Comparing posthypoxia levels of OCR in retina vs. islets (Figure 5), islets did not recover from hypoxia as well as retina suggesting that the approach can be used to assess sensitivity to the stress of ischemia-reperfusion conditions. Note that due to the delay in time it took for the inflow perfusate to reach a new equilibrium, the inflow O_2_ did not reach equilibrium levels with the O_2_ from the supply gas tank. The levels of O_2_ in the outflow are dependent upon the inflow O_2_, the flow rate, and the OCR of the tissue in the chamber. In order to match the levels of O_2_ that each tissue was exposed to, the supply gas tanks used for the hypoxia phase of the experiments were selected to generate similar outflow O_2_ levels for the retina (1% yielded an outflow of 6.5 mm Hg) vs. islets (3% yielded 5.5 mm Hg).

In response to hypoxia, lactate and pyruvate production rates by retina increased, consistent with operation of the Pasteur effect. The ratio of lactate/pyruvate also increased during low O_2_ conditions (Figure 4B), reflecting the decreased uptake of pyruvate into the mitochondria, and the increase in the cytosolic redox state (NADH/NAD) that occurs during low O_2_ (Lai and Miller, 1973). These results provide support for the utility of measuring extracellular lactate and pyruvate for real-time responses to events affecting metabolism.

### Complex time- and concentration-dependent effects of H_2_S on ISR by islets resolved by fluidics analysis

**Figure 6.**
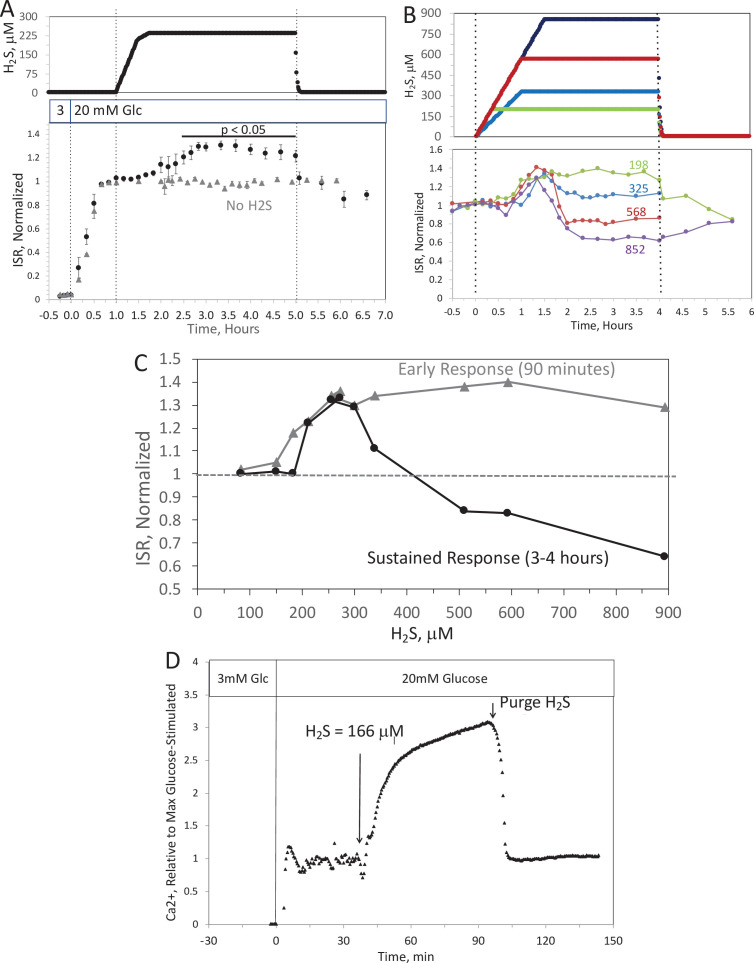
Effect of H_2_S on insulin secretion rate (ISR) by islets. (**A**) Rat islets (50/channel) were perifused (flow rate = 200 μl/min), and ISR was measured in response to glucose and exposure to dissolved H_2_S in the concentrations shown (data are average ± standard error [SE], *n* = 3 [H_2_S], *n* = 2 [no H_2_S], p < 0.05 as indicated). (**B**) ISR was measured at the indicated concentrations of dissolved H_2_S. Each curve is a single experiment. (**C**) Data from perifusions as shown in B were plotted as a function of the ISR at the peak between 1 and 1.5 hr, and the average ISR between 3 and 4 hr. (D) Response of cytosolic Ca^2+^ to changes in glucose concentration and exposure to 166 μM H_2_S and its washout. Raw data can be found in a Source Data file named ‘Figure 6—source data 1’. Figure 6—source data 1.Effect of hydrogen sulfide on insulin secretion rate.

Past studies on the effect of H_2_S on ISR were consistent in their findings that H_2_S was inhibitory (Niki and Kaneko, 2006; Wu et al., 2009; Yang et al., 2005; Bełtowski et al., 2018; Tang et al., 2013; Lu et al., 2019; Patel and Shah, 2010; Yang et al., 2007). However, as H_2_S has both stimulatory and inhibitory effects on the ETC (Khan et al., 1990; Vitvitsky et al., 2018), we predicted that precise titration of the exposure of islets to H_2_S would reveal stimulatory effects of H_2_S on ISR. To simplify the analysis and interpretation, we ramped up the H_2_S concentration in the gas equilibration system (while the inflow and outflow gas ports were clamped) to accumulate H_2_S until the desired concentration was reached, and then clamped the permeation tube inlet port to maintain that H_2_S concentration for the indicated times. When H_2_S was increased until a steady state of 240 μM was reached, ISR from pancreatic islets increased by 35% relative to ISR at 20 mM glucose (Figure 6A). The increased ISR was sustained for 3 hr. The effect of H_2_S was reversible. After purging it from the system, ISR rapidly returned to levels that occurred prior to H_2_S exposure. In the presence of 3 mM glucose, H_2_S had no effect on ISR (data not shown), supporting the idea that this reflects a physiologic response of ISR to H_2_S. To demonstrate the ability of the flow system to more fully characterize the time- and concentration dependency of ISR on H_2_S, we measured ISR at steady-state concentrations of H_2_S from 80 to 780 μM. Notably, between 180 and 780 μM H_2_S (Figure 6B), the initial period of stimulation of ISR (peaking between 1 and 1.5 hr after the start of the ramp of H_2_S) was insensitive to the concentration of H_2_S. In contrast, the effect of higher levels of H_2_S inhibited the ISR rate only between 1.5 and 4 hr following the start of the H_2_S exposure. The initial upslope of ISR occurred with a delay of about 30 min, and both the stimulation and inhibition of ISR were rapidly reversible following the washout of H_2_S (Figure 6B). The ability of the system to resolve the time lag for H_2_S to activate ISR is limited by the rate of the increase of H_2_S in the gas equilibration system accomplished by the permeation tube. In order to increase the temporal resolution, the permeation tube leak rate can be increased, or the volume of the gas equilibration system must be decreased. Additional concentrations of H_2_S were tested and the observed peak of ISR, and the steady-state level between 3 and 4 hr were plotted as a function of the concentration of H_2_S (Figure 6C), clearly showing the ability of the system to resolve both the time courses and concentration dependency of the effects of a relatively small range of [H_2_S]. In order to demonstrate the ability of the system to investigate mechanisms mediating the effects of H_2_S on ISR, we subsequently measured intracellular Ca^2+^ in response to H_2_S at a concentration that increased ISR (Figure 6D). Consistent with a stimulatory effect of H_2_S on ISR, intracellular Ca^2+^ increased dramatically upon exposure to H_2_S, and returned to near pre-H_2_S levels within a few minutes of washing out the H_2_S.

To test the assumption made in many studies that due to rapid equilibrium between HS^−^ and dissolved H_2_S, NaHS is able to emulate direct exposure to H_2_S (Li and Lancaster, 2013), we also analyzed the effect of NaHS on ISR and Ca^2+^ by perifused islets. In contrast to dissolved gaseous H_2_S, low levels of NaHS had no effect, and higher concentrations (>1 μM) inhibited ISR (Appendix 1—figure 1A, B) – consistent with findings of all previous studies that used NaHS as an H_2_S surrogate (Niki and Kaneko, 2006; Wu et al., 2009; Yang et al., 2005; Bełtowski et al., 2018; Tang et al., 2013; Lu et al., 2019; Patel and Shah, 2010; Yang et al., 2007). Similarly, NaHS did not increase Ca^2+^, and slightly decreased it by amounts that were relatively insensitive to the concentration of NaHS (Appendix 1—figure 1C). The inhibitory effects of HS^−^, which is present at about double the concentration of dissolved H_2_S, may in part account for the complex concentration dependency seen in Figure 6B, C. The likely explanation of the difference between effects of H_2_S and NaHS is that the H_2_S generated by NaHS diffuses out of solution and into the gas phase when the media is in contact with a headspace that does not contain gaseous H_2_S. To test this, 10 ml of KRB were placed in a sealed 125 ml bottle, and measured H_2_S in the KRB and the headspace as a function of time after either injecting NaHS into the solution or permeating gaseous H_2_S into the headspace (Appendix 1—figure 2A, B). The H_2_S in solution emanating from the headspace was higher than that achieved from NaHS, but notably it declined when the headspace was purged. Although the amount in the headspace was close to the detection limit of the H_2_S measurement method, it was clear from the sharp decline in H_2_S after unsealing the bottle, that H_2_S from the NaHS had indeed transferred into the headspace. Thus, these data support a scenario where differences in effects of H_2_S and NaHS occur due to the absence of H_2_S in solution containing NaHS and HS^−^ is inhibitory for both ISR and Ca^2+^.

### Measurement of OCR, cytochrome c, lactate, and pyruvate by perifused liver slices

We also explored the ability of our flow system to measure effects of H_2_S on liver by measuring OCR, cytochromes, and lactate/pyruvate release by liver slices in the absence and presence of a mitochondrial fuel (succinate). The responses were complex, changed directions in time- and concentration-dependent fashion, and will ultimately require more experiments to interpret the data mechanistically. Therefore, these data were placed in the appendix (Appendix 1—figures 3 and 4). Nonetheless the waveforms of the responses were clearly resolved, confirmed the capabilities of the multiparametric detection system, and so were included in this report. The salient features of the data can be summarized by: (1) In the absence of the mitochondrial fuel succinate, H_2_S changed OCR and reduced cytochromes in proportion to each other, consistent with donation of electrons from H_2_S to cytochrome c (Vitvitsky et al., 2018; Appendix 1—figure 3); (2) In the presence of a TCA cycle intermediate (succinate), H_2_S (200–300 μM) increased the reductive state of cytochrome c oxidase while decreasing OCR. This is consistent with inhibition of cytochrome c oxidase (Khan et al., 1990; Appendix 1—figure 4), which occurred at concentration similar to typical estimates of plasma concentration which range from 30 to 300 μM (Olson, 2009). At low levels, H_2_S caused irreversible inhibition of ETC activity upstream of cytochrome c but did not inhibit flow of electrons from succinate. Thus, both reported mechanisms of action of H_2_S on the ETC were resolved by the system, as well as uncovering additional effects of H_2_S that had not previously been reported.

## Discussion

### General features of the flow system

Flow systems have important advantages over static systems for assessment of cell function. Viability and functions of cells and tissues are better and closer to physiological, culture media composition can be changed, and real-time production or uptake rates can be quantified from differences between inflow and outflow. Microfluidics devices can maintain tissues in ways that preserve their three-dimensional structure and preserve native cell-to-cell interaction (Nieskens and Wilmer, 2016; Rothbauer et al., 2018). However, these devices will have maximal impact when combined with real-time assessment of the tissue as well as the ability to control aqueous and gaseous composition of the media bathing the tissue models. This report focuses on technical modifications to a previously developed flow culture/assessment system (Sweet et al., 2004) that enables real-time measurements of responses of tissues to physiologically important dissolved gases. We achieved this by incorporating a unique gas equilibration system that controls abundant (blood) gases including O_2_, CO_2_, and N_2_, and by using permeation tubes to introduce and control endogenously-produced gases such as H_2_S, NO, and CO. In this report, we demonstrated the utility of this system using both a blood (O_2_) and a signaling gas (H_2_S).

### Control and effects of dissolved O_2_: Recovery of metabolic state following hypoxia and transient response in OCR following reoxygenation

The ability to control dissolved O_2_ makes our system highly suitable for investigating ischemia–reperfusion injury, generating two informative endpoints from a protocol that measures effects of a short period of low O_2_ availability followed by return to normal levels. We used the recovery of OCR and cytochrome c reduction to report tissue sensitivity to hypoxia; and we identified a transient spike in OCR that occurs upon reintroduction of normal O_2_ levels.

The first endpoint characterizes the capacity of a tissue to survive after exposure to selected time periods of low O_2_. In the illustration carried out in this study, retina recovered to 83% after hypoxia, whereas islets recovered to only 55% of prehypoxic levels of OCR, corresponding to the two tissue’s known sensitivity to oxidative stress. The second endpoint, the burst of OCR occurring when O_2_ floods back into the cell, is one that has been hypothesized, but has not previously been measured due to the difficulty of measuring OCR in the face of changing O_2_ levels. Our method has enabled the measurement of transient responses to reoxygenation and revealed a two-phase waveform in OCR in both islets and retina. The ability to resolve this waveform was dependent on rigorous convolution analysis to remove the delay and dispersion of the O_2_ signal due to the flow system, combined with ultra-stable and ultra-sensitive O_2_ sensors. It has been long recognized that ROS is generated rapidly by cells when O_2_ becomes plentiful after undergoing hypoxic conditions (Chouchani et al., 2016; Zweier et al., 1987). The transient spike of OCR is consistent with a scenario where O_2_ is the source of oxygen atoms for the ROS. This is also consistent with the buildup of metabolites such as succinate during ischemia (Beach et al., 2020), which could fuel increased oxidation rate. The detailed relationship between OCR and generation of ROS will be the topic of future applications with this system. For instance, the method can be used to test whether slowed reintroduction of O_2_ or candidate therapeutics including H_2_S, prevent or reduce the transient spike in OCR while at the same time preventing the decreased recovery following the hypoxic period (as has been hypothesized; Clark and Gewertz, 1992; Zhang, 2020; Citi et al., 2018; Du et al., 2017). Other applications could include: testing whether there are differences in transient OCR response for different tissues or metabolic states; determining the relation of the spike to the response of ROS and recovery/survival of tissue; and testing drugs designed to prevent both the transient spike and/or the decrease in posthypoxic OCR. The ability to objectively quantify recovery of OCR positions the system to be used to test tissue sensitivity to a wide range of stresses (including ER and oxidative stress, immunological stress [exposure to cytokines], lipotoxicity as well as hypoxia), and to test drugs and treatments designed to increase or decrease recovery from experimentally induced stresses.

### Control and effects of a trace gas: stimulation of ISR and Ca^2+^ by H_2_S in islets

Gas signaling molecules (CO, NO, and H_2_S) are generated in most tissues and have wide-ranging effects on function, metabolism, and protection from hypoxia (for reviews Ahmad et al., 2018; Beltowski, 2007; Cheng and Rong, 2017; Krylatov et al., 2021). However, due to the difficulty in quantitatively and reproducibly introducing dissolved trace gases into culture media, the majority of studies on these gases have utilized aqueous chemical donors such as NaHS instead of H_2_S. These surrogates provide only imprecise and uncertain concentrations and timing of tissue exposure to dissolved H_2_S. Due to the extremely accurate calibration of the rate of release of gases, permeation tubes can introduce trace gases into the carrier gas (the mixture of CO_2_–O_2_–N_2_) present in a gas equilibration system of our perifusion apparatus at exact times and concentrations. Producers of permeation tubes can provide them with a selection of over 500 gases, including H_2_S, NO, CO, and ammonia. Thus, the method of incorporating permeation tubes into the gas equilibration system is particularly versatile and can be used for a wide range of applications.

To illustrate the unique advantages of being able to expose tissue to precise levels of dissolved H_2_S, we selected islets as a test tissue since the inhibitory effects of H_2_S on glucose-stimulated ISR and cytosolic Ca^2+^ by islets have been described. However, those reports were based on use of the H_2_S donors NaHS and Na_2_S (Niki and Kaneko, 2006; Wu et al., 2009; Yang et al., 2005; Bełtowski et al., 2018; Tang et al., 2013; Lu et al., 2019; Patel and Shah, 2010; Yang et al., 2007) and the interpretation that H_2_S gas is actually delivered to the tissue and at levels low enough to avoid its toxic effects. Moreover, we took note of other studies that suggested that H_2_S can donate electrons directly to cytochrome c (Vitvitsky et al., 2018) and there is evidence that the reduction of cytochrome c may be a key regulatory step in activating ISR (Rountree et al., 2014; Jung et al., 2011). Our system bore out this prediction revealing stimulatory effects of H_2_S on ISR that had not been apparent when exposing islets to a donor of H_2_S (NaHS). H_2_S at concentrations (between 140 and 280 μM) enhanced glucose-stimulated ISR, which remained elevated for at least 4 hr. The concentration- and time dependency were complex however. At higher concentrations of H_2_S the stimulation of ISR for 90 min still occurs, but at later times ISR was inversely proportional to the H_2_S ranging from a 35% increase to a 40% decrease. The U-shaped concentration dependency may also reflect the effects of both H_2_S and inhibitory effects of HS^−^. At low concentrations NaHS had little effect on ISR or cytosolic Ca^2+^, however as its concentration approached 1 μM and above, both endpoints were inhibited. The range of effects occurred over a relatively small range of H_2_S concentrations, highlighting the need for the very precise control of dissolved H_2_S afforded by the use of permeation tubes to investigate this phenomenon. H_2_S had no effect on ISR at 3 mM glucose, supporting a physiologic mechanism mediating H_2_S’s effect that may be integrated with glucose sensing and secretory response to fuels by the islet (Prentki et al., 2013; Campbell and Newgard, 2021).

The stimulatory effects of H_2_S on ISR and Ca^2+^ directly refute the well accepted conclusions that H_2_S decreases ISR and Ca^2+^ by opening K_ATP_ channels as has been widely reported (Ali et al., 2007; Yang et al., 2005; Lu et al., 2019; Shoji et al., 2019). Although it is typically assumed when using NaHS as a source of H_2_S that NaHS and H_2_S equilibrate in solution (Olson, 2009), our measurements show that in the absence of H_2_S in the headspace above the media, dissolved H_2_S quickly disappears into the headspace, a phenomenon analogous to the behavior of CO_2_-based buffers. Thus, measurements of effects of NaHS on tissue in open systems such as typical static and perifusion methods contain little H_2_S. Thus, our method offers a novel and uniquely capable approach for investigating the direct effects of the protonated form of H_2_S in equilibrium with HS^−^.

H_2_S is generated in islets by the action of three intracellular enzymes (Kimura, 2010) but is also a component of blood albeit at levels that are not well established (Whiteman et al., 2010; Karunya et al., 2019; Whitfield et al., 2008). It is notable that the range of concentrations that induced changes in ISR, and above which caused inhibition of OCR in liver are in the range of typical estimates of plasma concentration which range from 30 to 300 μM (Olson, 2009). Therefore, it suggests that intracellular effects of H_2_S could be mediated by H_2_S derived from the blood as well as H_2_S endogenously produced by cells. The ability to detect differences between responses to H_2_S and donor molecules, will be useful to the increasing numbers of investigators developing H_2_S donor molecules as pharmaceutics (Citi et al., 2020; Testai et al., 2020). The increase in ISR in response to H_2_S has physiological, methodological, and clinical implications and the lack of similar stimulatory effects by a donor molecule has broad implications in a field where studies of NO, H_2_S, and CO are mostly based on the use of donor molecules.

### Lactate and pyruvate: relation to cytosolic events

The assayed values of lactate and pyruvate reflect a number of important specific and global parameters. The rate of release of lactate and pyruvate is an integration of the rate of glycolysis less the amount of pyruvate flux into the mitochondria and traversing gluconeogenesis. Thus, both compounds generally increase in response to glycolytic fuels. Importantly for the study of hypoxia, both metabolites rise in cells when O_2_ is decreased (the Pasteur effect). In addition, the ratio of cytosolic lactate/pyruvate mirrors the cytosolic NADH/NAD ratio due to the equilibrium status of the LDH reaction (Newsholme and Start, 1973). One could envision that freeze clamping cells and measuring intracellular lactate and pyruvate to directly compare them to the extracellular values could be a way to validate the use of extracellular data. However, in practice, the measurement of intracellular compounds is difficult and also limited by the kinetic resolution of freeze clamping. Instead, we evaluated the responses of the extracellular levels of lactate to a blocker of LDH (oxamate), of pyruvate to a blocker of mitochondrial transport (zaprinast), and both compounds in response to hypoxia. The rapid changes in extracellular lactate and pyruvate support the rapid redistribution between intra- and extracellular compartments, and that real-time measurement of extracellular lactate and pyruvate reflect intracellular events governing intracellular lactate and pyruvate. Retina responded to hypoxia with a classical Pasteur effect: low O_2_ increased lactate, pyruvate, and lactate/pyruvate ratio. We envision that the measurement would be especially informative when examining the shift from oxidative to glycolytic metabolism such as seen in tumorigenesis (de Groof et al., 2009) or stem cell differentiation (Zhou et al., 2012).

### Incorporation of O_2_ control into a real-time fluorescent imaging system

Real-time fluorescent imaging is a powerful modality that is commonly used to quantify a wide variety of intracellular compounds and factors while perfusing the optical chamber housing the cells or tissue. Molecular probes provide intracellular dyes for over 100 separate compounds, so this method is versatile and wide ranging. Thus, incorporating the gas equilibration system to a flow system providing buffer to a chamber that images of single islets. We observed and quantified clear increases in intracellular Ca^2+^ in response to hypoxia as metabolic rate decreased. Unexpectedly, the loss of energy and ISR following islet exposure to hypoxia were not accompanied by a loss of glucose-stimulated Ca^2+^, suggesting the mechanism mediating loss of ISR is independent of Ca^2+^ signaling. These data are consistent with previous findings that loss of secretory function is more closely associated with bioenergetics than Ca^2+^ (Rountree et al., 2014; Rountree et al., 2013). When comparing results of various assays and modes of analysis, the ability to measure multiple endpoints under matched conditions and the same flow system is optimal for systematic study of tissue function.

### Summary of uses for the flow culture system

The ability to precisely control the levels and timing of exposure to both abundant and trace gases while measuring multiple parameters in real time on a wide range of tissue and cell models make this system uniquely powerful. Although we illustrated the method using rodent tissue, it will have particular utility when maintaining and assessing human tissue. The novel resolution of OCR transients attests to the high kinetic resolution of the system. Moreover, stimulatory effects of H_2_S on ISR and Ca^2+^ not seen in response to donor molecules, attests to the ability of the technology to reveal behavior that provides new insight. Given the wide use of donor molecules in studying gasotransmitters this has broad and significant implications. In addition to enabling users to evaluate direct effects of gases, the method is also suitable for testing conditions or agents that diminish loss of OCR in response to hypoxia- or other stress-induced effects. The use of methods to study the effects of dissolved gas on tissue will impact many areas of fundamental research as well as research of diseases including but not limited to diabetic wound healing, stroke (ischemia/reperfusion injury), and cancer.

## Materials and methods

### Chemicals

Krebs–Ringer bicarbonate buffer was used for all perifusions prepared as described previously (Neal et al., 2015). Antimycin A, glucose, oxamate, KCN, and zaprinast were purchased from Sigma-Aldrich. Gases of varying O_2_ levels/5% CO_2_ and balance N_2_ were purchased from Praxair Distribution Inc (Danbury, CT). Cytodex and Cytopore beads were purchased from GE healthcare and biosciences (cat no. 17-0448-01 and 17-0911-01, respectively).

### Culture of INS-1 832/13 cells

INS-1 832/13 cells were kindly provided by Dr. Christopher Newgard (Duke University) who initially generated the cell line (Hohmeier et al., 2000). Their identity was confirmed by a variety of functional tests that reveal characteristics unique to this beta cell line (increase in OCR, intracellular calcium, lactate production, and insulin secretion rate in response to physiological changes in glucose [5–10 mM glucose] that are intrinsic to beta cells). Tests for mycoplasma contamination in the cell suspension were negative. Cells were grown and cultured as previously described (Hohmeier et al., 2000). The day before experiments, cells were harvested, and cultured with Cytodex beads (2.5 mg/million cells) for 15 min in RPMI Media 1640 (Gibco, Grand Island, NY) supplemented with 10% heat-inactivated fetal bovine serum (Atlanta Biologicals, Lawrenceville, GA), 2 mM L-glutamine, 1 mM pyruvate, 50 μM beta-mercaptoethanol, 20 mM HEPES, and 1 % Pen/Strep. They were then washed and cultured overnight in a standard CO_2_ incubator at 37°C.

### Tissue harvesting and processing

All procedures were approved by the University of Washington Institutional Animal Care and Use Committee.

#### Rat islet isolation and culture

Islets were harvested from male Sprague-Dawley rats (approximately 250 g; Envigo/Harlan, Indianapolis, IN) anesthetized by intraperitoneal injection of sodium pentobarbital (150 mg/kg rat) and purified as described (Sweet et al., 2004; Matsumoto et al., 1999). Subsequently, islets were cultured for 18 hr in RPMI Media 1640 supplemented with 10% heat-inactivated fetal bovine serum (Invitrogen) at 37°C prior to the experiments.

#### Retina isolation

Retinas were harvested from C57BL/6J mice (euthanized by cervical dislocation) 10 min prior to loading and were dissected into ¼ths using microscissors as previously described (Bisbach et al., 2020).

### Flow culture system to maintain tissue with precise control of dissolved gases

**Figure 7.**
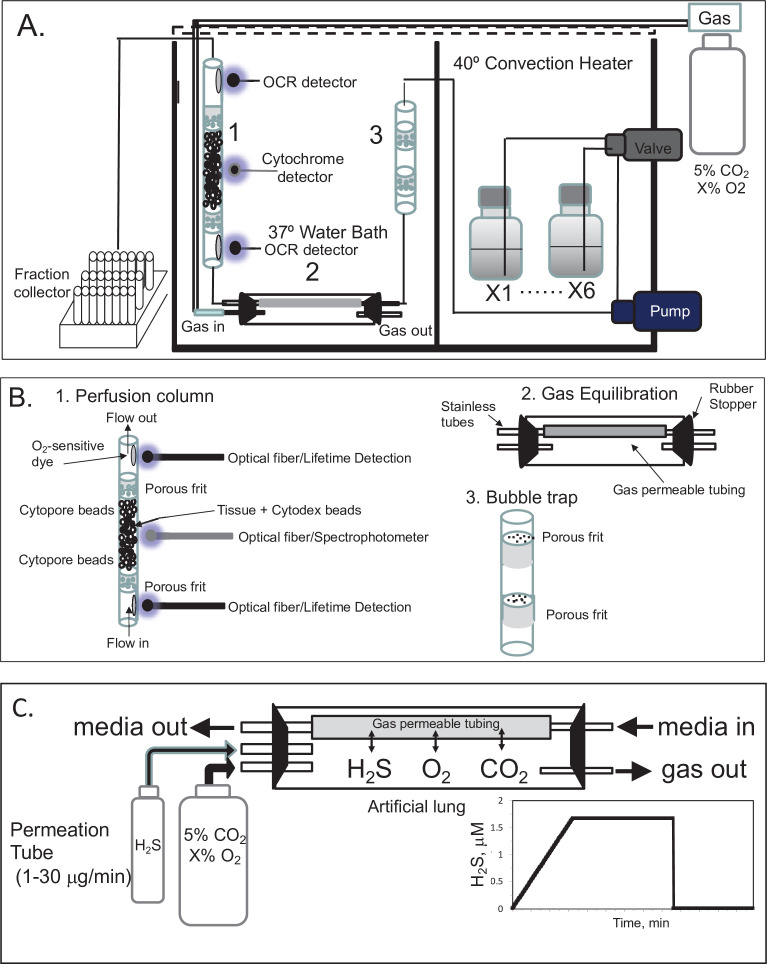
Flow culture system/assessment system for studying effects of dissolved gases on tissue or cells. One channel/perifusion chamber is shown, but the actual system can accommodate up to 8. (**A**) The perifusion system consisted of an eight-channel peristaltic pump (MiniPuls 2, Gilson, Middleton, WI) connected to a six-port valve (Part # V-451, IDEX Health and Science, Oak Harbor, WA) to produce up to six separate solutions; a media/dissolved gas equilibrium system in which media flowed through thin-walled silastic tubing (0.062 in ID × 0.095 in OD; Dow Corning Corp, Midland, MI) for a residence time of 5 min (typically 0.2–0.5 m depending on the flow rate) loosely coiled in a glass jar that contained various O_2_, 5% CO_2_/balance N_2_; bubble trap comprised of a Simax Borosilicate glass tube (Mountain Glass, Asheville, NC, 2″ long and 4.2 mm ID) filled with glass wool; a Simax Borosilicate glass perifusion chamber (3″ long and 4.2 mm ID) immersed in a 37°C water bath; Lifetime detection spectrometers (Tau Theta, Boulder CO), a tungsten-halogen light source/USB2000 spectrophotometer (Ocean Optics OH); and a Foxy 200 fraction collector (Isco, Inc, Lincoln, NE). (**B**) A blow up of individual parts shown in A. (1) The glass perifusion chamber containing culture beads, porous frits (Interstate Specialty Products, Suton, MA, Cat no. POR 4894, cut to 4.2 mm diameter and 0.25 in long) to support the tissue and disperse the flow, and coated with O_2_-sensitive dye on the interior above and below where the tissue resides; (2) gas equilibration chamber, where media flows through gas-permeable silastic tubing and equilibrates with the gases filling the headspace; (3) bubble traps. (**C**) Incorporation of a permeation tube (VICI Metronics, Poulsbo, WA) that releases H_2_S at specified rates into the media/dissolved gas equilibration system during which time the ports for the inflow and outflow of carrier gas (O_2_, CO_2_, and N_2_) are closed. The resulting accumulation of H_2_S in the artificial lung yields linearly increasing concentrations of dissolved H_2_S in the form of a ramp function as shown.

A flow culture system (Neal et al., 2015) was modified to continuously perifuse tissue with buffer equilibrated with the desired composition of dissolved gas using a gas equilibration system (Figure 7A, B). Multiple modes of assessment were integrated into the flow culture system and are described below including chemical sensors for O_2_, spectroscopic analysis of the tissue for measurement of reduced cytochrome c and cytochrome c oxidase, and collection of outflow fractions for subsequent assay of lactate and pyruvate, or insulin. Model numbers and manufacturers are listed in the legend for Figure 7. Prior to entering the perifusion chamber, perifusate is pumped from the media reservoirs by an eight-channel peristaltic pump into the thin-walled silastic tubing of the gas equilibration system that facilitated equilibration between the buffer and gas in the glass housing. The use of the gas equilibration system avoids the issue of outgassing of dissolved gases (DeLeon et al., 2012) from the perifusate prior to flowing past the tissue and sensors. Selection of media reservoir with desired media composition determined by use of a six-port valve. To achieve the desired gas composition of O_2_, CO_2_, and N_2_ within the gas equilibration system, tanks of premixed gases supplied gas to the inflow port, typically 5% CO_2_, the desired percentage of O_2_, and balance N_2_.

To equilibrate the inflow with desired concentration of H_2_S, we have used devices called permeation tubes (VICI Metronics, Poulsbo, WA), an industry standard that provides very precise rates of gas release, typically from 1 to 30 μg/min. The outlet of the permeation tube was connected to the inlet port of the chamber housing the gas equilibration system (Figure 7C), so that the headspace around the perifusate in the gas-permeable tubing accumulated the trace gas. The concentration in the chamber then increased as a ramp function, rising at a rate equal to the leak rate of the permeation tube x time divided by the volume of the housing. Henry’s constant is defined as

$$Hc=1000\times [gas{]}_{aq}/[gas{]}_{g}$$

where [gas]_aq_ is in μM, and [gas]_g_ is in ng/ml.

At 37 degrees dissolved O_2_ is 217 nmol/mL in KRB, which is in equilibrium with 0.3008 mg/mL of O2 in air (21%). Therefore,

$$Hc(O_{2})=1000\times [O_{2}{]}_{aq}/[O_{2}{]}_{g}=217\mu M/300,800ng/mL\times 1000=0.721$$

In order to estimate Henry’s constants for H_2_S at 37 degrees in KRB, we used measurement of solubility (reported by National Institute of Standards and Technology) normalized relative to O_2_ in the relationship

$$Hc(H_{2}S)=Solubility(H_{2}S)/Solubility(O_{2})\times Hc(O_{2})=0.1/0.0013\times 0.721=55.5$$

Thus, the equation relating the dissolved H_2_S concentrations to head space concentration is:

(1) $$[H_{2}S_{aq}]=[H_{2}S_{g}]\times \;\;Hc/1000$$

where [H_2_S_aq_] is in μM, and [H_2_S_g_] is in ng/ml.

#### Lifetime detection of dissolved O_2_

**Figure 8.**
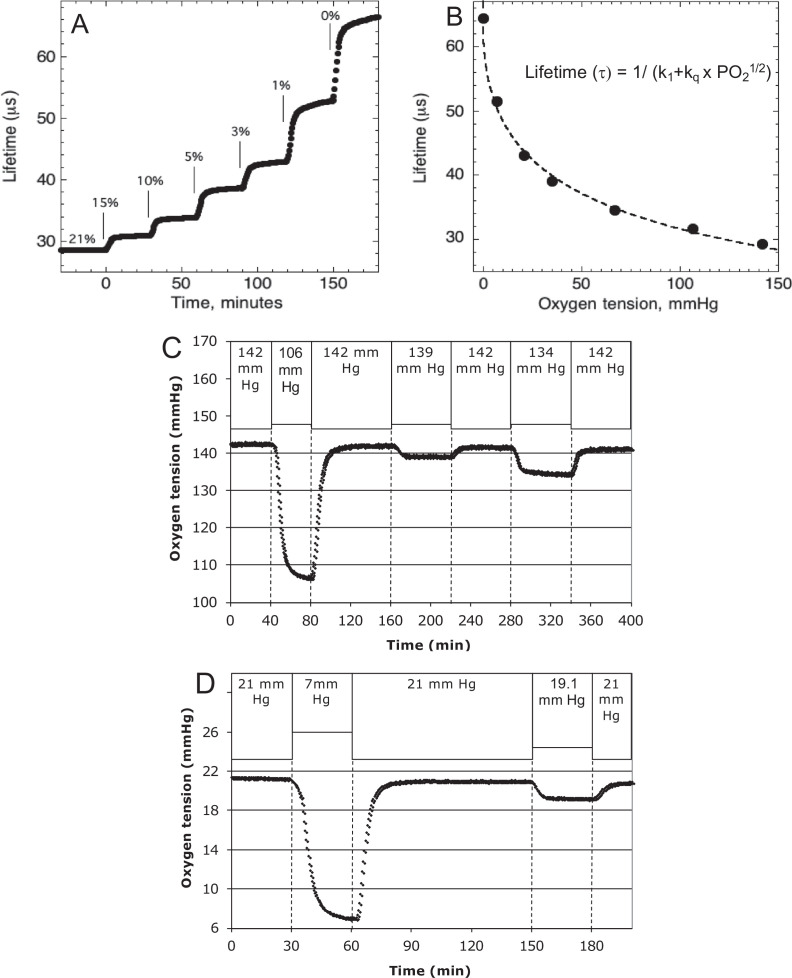
Control and measurement of dissolved O_2_. (A) At 20- to 30-min intervals, the lung was sequentially filled with 21%, 15%, 10%, 5%, 3%, 1%, and 0 % O_2_. (**B**) The steady-state lifetime measurements were nonlinear and conformed to a Stern–Volmer equation. Where the pO_2_ is partial pressure of oxygen molecules (oxygen tension), *τ*_0_ is lifetime of the dye without quencher such as oxygen molecule, *k*_1_ is 1/*τ*_0_ and *k*_q_ is a bimolecular quenching constant of the dye by oxygen molecules. The *k*_1_ and *k*_q_ were 15,519 s^−1^ and 1612.2 mm Hg^−1^ s^−1^, respectively. (**C**) Test of precision at normal O_2_. O_2_ (142 mm Hg) in the lung was changed first to 106.5 mm Hg and back to 142 (levels of change that were typical what was observed in our studies). By mixing 21% and 15% tanks at known flow rates, O_2_ was then decreased by 3 mm Hg and then subsequently by 8 mm Hg. (**D**) Test of precision and S/N of low O_2_. O_2_ (21 mm Hg) in the lung was changed first to 7 mm Hg (1% O_2_) and back to 21. By mixing 3% and 1% tanks at known flow rates, O_2_ was decreased by 1.9 mm Hg. S/N was for the 14 and 1.9 mm Hg changes was >80 and >10, respectively. Raw data can be found in a Source Data file named ‘Figure 8—source data 1’. Figure 8—source data 1.Control and measurement of dissolved O_2_.

O_2_ tension in the inflow and outflow buffer was measured by detecting the phosphorescence lifetime of an O_2_-sensitive dye painted on the inside of the perifusion chamber using a MFPF-100 multifrequency phase fluorometer lifetime measurement system (TauTheta Instruments, Boulder, CO) as previously described (Sweet et al., 2002). Using tanks of gas containing varying amounts of O_2_ (21%, 15%, 10%, 5%, 3%, 1%, or 0%), data were generated that showed the dependency of the lifetime signal as a function of O_2_ and the rapidity of changes in O_2_ after each change in gas tank. Within 5 min, O_2_ achieves 95 % of steady-state levels (Figure 8A), where the delay is primarily due to time needed for the gas in the gas equilibration system to turnover as the actual sensor responds in microseconds. The O_2_ dependency of the dye signal conformed to the Stern–Volmer equation.

(2) $$Lifetime=1/(k_{1}+k_{q}*[O_{2}{]}^{1/2})$$

where lifetime is in µs. Equation 2 was used to as a calibration curve to convert the optical signals to O_2_ content (Figure 8B). The use of lifetime detection produces very stable and sensitive data at both normal (Figure 8C) and low (Figure 8D) O_2_ levels producing S/N over 20 even when measuring a change of only 1.9 mm Hg.

### Continuous measurement of OCR

#### Measuring the difference between inflow and outflow during invariant inflow O_2_

When inflow O_2_ tension is constant, OCR by the tissue equals the difference between the content of O_2_ flowing into the perifusion chamber minus that flowing out time the flow rate as follows.

(3) $$OCR=([O_{2}]in-[O_{2}]out)\times flowrate$$

where flow rate is in μl/min and [O_2_] is in nmol/ml. Inflow and outflow O_2_ sensors were positioned on the inside of the perifusion chamber 2 cm upstream and 2 cm downstream from the tissue, respectively. Perifusate flow rates were set to result in a difference between inflow and outflow O_2_ of between 5% and 25% of the inflow O_2_ signal, so it was large enough to be accurately measured, but small enough to avoid exposure to unintended hypoxic conditions.

#### Measuring OCR during changes in inflow O_2_: convolution analysis to remove system effects

**Figure 9.**
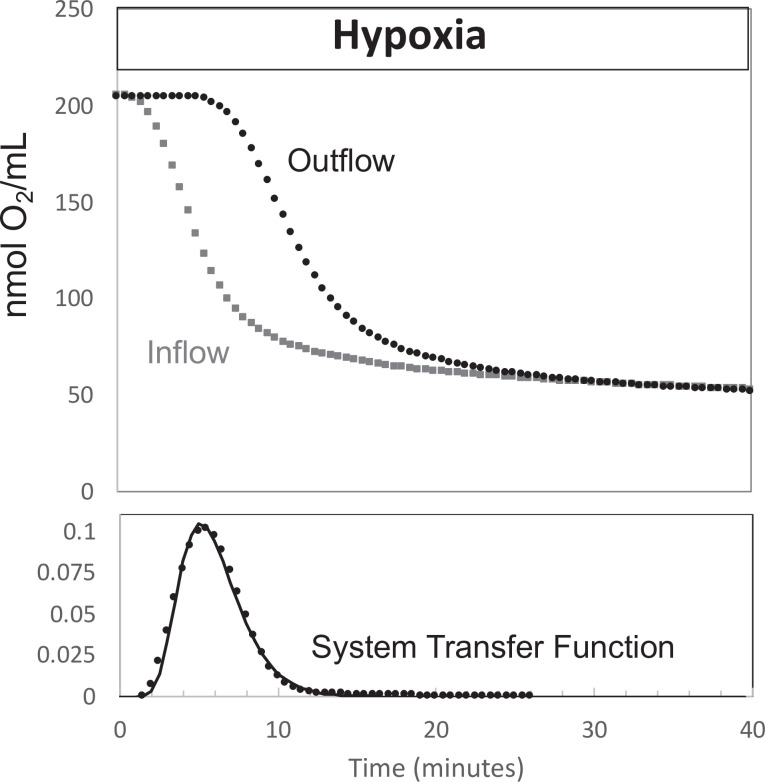
Determination of transfer function from inflow and outflow for convolution analysis. Measurement of inflow and outflow O_2_ tensions in response to a change from 21% to 3% with no live tissue in the system. Deconvolution was carried out to generate the transfer function of the system shown on the bottom graph. Raw data can be found in a Source Data file named ‘Figure 9—source data 1’. Figure 9—source data 1.Determination of transfer function from inflow and outflow data.

Measuring the temporal changes in OCR by tissue in the face of changing inflow concentrations of O_2_ requires a correction for the difference in inflow and outflow O_2_ levels due to the delay and dispersion generated between the inflow and outflow sensors. To calculate OCR from Equation 3 in the face of changing inflow O_2_, the inflow O_2_ content must be converted to what it would be if the sensor was located at the outflow sensor location. This was done with classical convolution methods (Weigle et al., 1987) with mild regularization (Bube and Langan, 2008) to create a mathematical function representing the delay and dispersion of the inflow signal by the flow through the perifusion chamber from the inflow to the outflow sensor described numerically by Equation 4.

(4) $$\left[O_{2}{]}_{in:transformed}=[O_{2}{]}_{in}*h(t\right)$$

where [O_2_]_in:transformed_ is the inflow concentration at the outflow sensor, and *h*(*t*) is the system transfer function. In the absence of tissue in the flow system, O_2_ was decreased to hypoxic levels in the same protocol as was done in the presence of tissue, while measuring [O_2_] in the inflow and outflow (Figure 9). The transfer function *h*(*t*) was then generated for each experimental condition by solving Equation 4 by deconvolution using MatLab. For each perifusion analysis, the measured [O_2_]_in_ was converted to [O_2_]_in:transformed_ by convolution with the transfer function (also using MatLab) and OCR was calculated from.

(5) $$OCR=([O_{2}{]}_{in:transformed}-[O_{2}{]}_{out})\times FR$$

For the protocols in this experimental setup, only 35 min of the transfer function was needed to accurately transform the inflow [O_2_].

### Measurement of cytochrome c and cytochrome c oxidase reduction

The reductive states of cytochrome c and cytochrome c oxidase were quantified by measuring spectra of light transmission from 400 to 650 nm through the column of islets or tissue as previously described (Chance and Williams, 1956a; Jung et al., 2011). From these spectral data, absorption, first and second derivatives were calculated as described below. In contrast to other methods used to measure changes in heme redox state in spinner systems (Kim et al., 2012; Kashiwagura et al., 1984), our use of a flow system allows continuous measurement of cytochromes in tissue for extended periods of time where the tissue is exposed to controlled media composition and no mechanical damage is inflicted on tissue by the spinner. Due to the low signal to noise and baseline shift during the experiments, direct measurement of absorption was not stable. To better resolve changes in the reduced state of cytochromes the second derivative of the absorbance spectra with respect to wavelength was calculated (Cavinato et al., 1990). Like absorption, this parameter reflects the number of electrons bound to the cytochrome as well as the amount of protein. However, the second derivative is unaffected by shifts in baseline allowing resolution of real-time changes in absorbance. At the conclusion of each experiment, calibration spectra for fully oxidized and reduced cytochromes were acquired in the presence of blockers of the ETC – namely 12 μg/ml antimycin to stop the flow of electrons to cytochromes, followed by 3 mM KCN to facilitate the maximal accumulation of electrons bound to cytochromes.

#### Spectral data processing

The second derivative of absorbance with respect to wavelength (Cavinato et al., 1990) at 550 and 605 nm was calculated (corresponding to cytochrome c and cytochrome c oxidase, respectively) as,

(6) $$Abs^{\prime \prime }=\frac{\Delta ⟮\frac{\Delta ABS}{\Delta V}⟯}{\Delta V}$$

where Abs = log (intensity − intensity_bkg_)/(intensity_ref_ − intensity_bkg_), *v* = wavelength in nanometers, and ∆ = change in the variable over the integration interval. Background intensity (intensity_bkg_) was determined with the light source off, and the reference intensity (intensity_ref_) was that obtained when cytochromes were fully oxidized by antimycin A. Percent reduction of cytochromes were calculated following Kashiwagura et al. (Kashiwagura et al., 1984) as,

(7) $${Cytochrome}_{red}=100\times \frac{{Abs}^{``}-{Abs}_{aA}^{``}}{{Abs}_{KCN}^{``}-{Abs}_{aA}^{``}}$$

where Abs″ and Abs″_KCN_ are values at 550 or 605 nm, and Abs″_KCN_ and Abs″_aA_ are obtained in the presence of KCN and antimycin A corresponding to when cytochrome c and cytochrome c oxidase are fully reduced or oxidized.

### Assays for lactate, pyruvate, and insulin

Fractions collected during the experiments were subsequently assayed for lactate, pyruvate, or insulin. Insulin was measured by RIA, and lactate and pyruvate were measured using colorimetric assays using kits per manufacturer’s instructions (insulin, Cat no. RI-13K, Millipore Sigma, Burlington, MA; lactate, Cat no. A22189, Invitrogen, Carlsbad, CA; pyruvate, Cat no. MAK332, Sigma-Aldrich). Amounts of lactate, pyruvate, and insulin in inflow samples were insignificant, so rates of production were calculated as the concentration in the outflow times the flow rate and normalized by the amount of tissue.

### Imaging and quantification of cytosolic Ca^2+^

Cytosolic Ca^2+^ was measured by fluorescence imaging of islets after loading them with Fura-2 AM (Invitrogen) as previously described (Jung et al., 2009). The perifusion system described above was used to supply buffer with the specified gas composition to a temperature-controlled, 250 μl perifusion dish (Bioptechs, Butler, PA) that was mounted on to the stage of a Nikon Eclipse TE-200 inverted microscope. Results are displayed as the ratio of the fluorescent intensities during excitation at two wavelengths (F340/F380).

### Statistical analysis

When the message to be conveyed by the graph was an illustration of the high resolution and low noise of the data that were generated by the method, then single experiments were shown as indicated, for instance for OCR in response to hypoxia. In most instances, to demonstrate the reproducibility of the data, technical replicates were conducted and the data averaged – that is multiple perifusion channels were run in parallel with pooled tissue or cells batches from multiple animals or flasks of cells. When the goal was to test and show a biological effect, multiple runs were done on different days (for instance comparison of retina and islet recovery of OCR following hypoxia) and statistical significance was determined using Student’s *t*-tests carried out with Microsoft Excel (Redmond, WA). With either technical or biological replicates, error bars on time courses were calculated as the average ± the standard error (calculated as SD/*n*^1/2^). Raw data for all experiments are compiled into Excel spreadsheets saved as a source file that is named with the same descriptor as the figure.

## Figures and Tables

**Appendix 1—figure 1.**
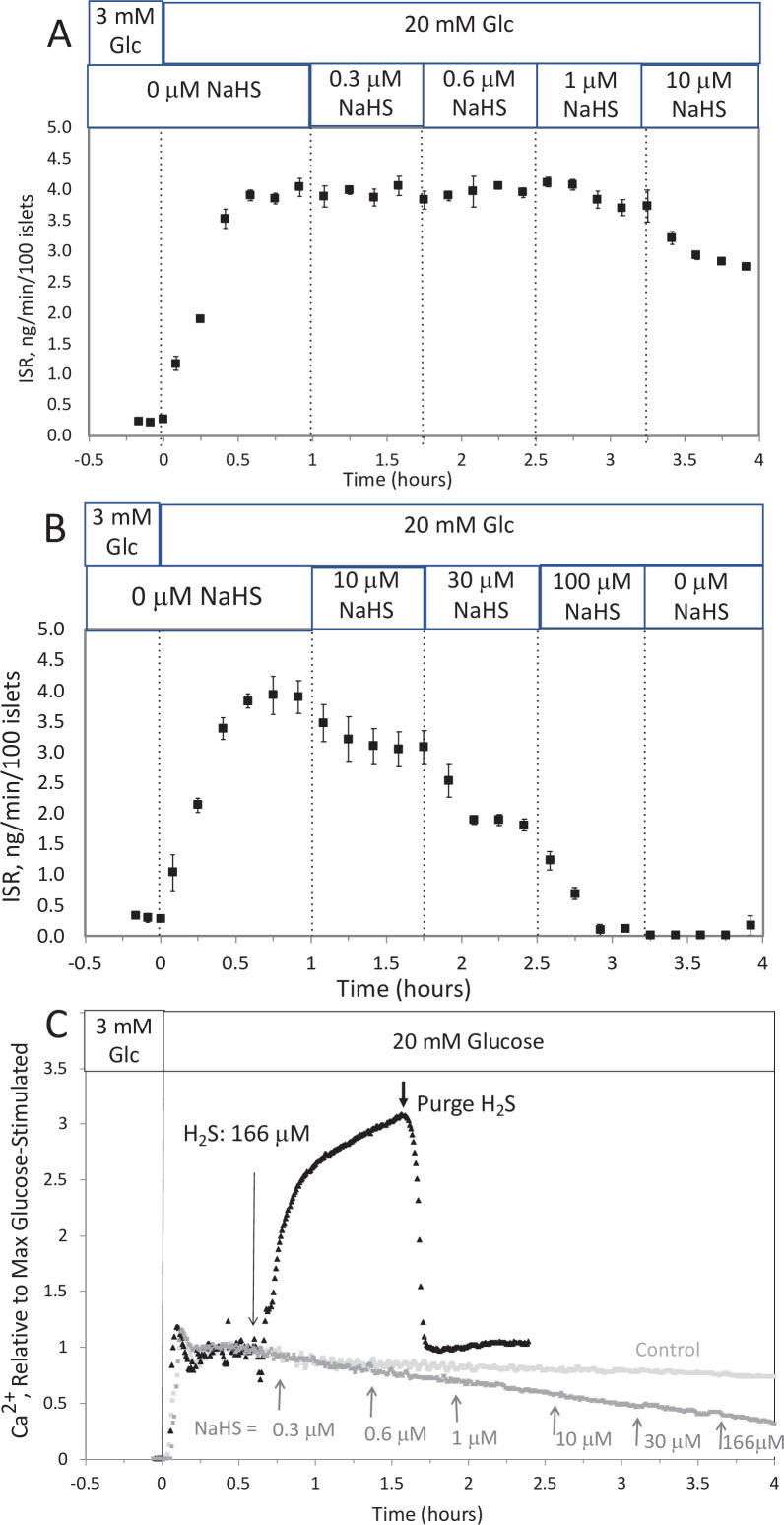
Effect of NaHS on insulin secretion rate (ISR) and cytosolic Ca^2+^ (A, B). Rat islets (50/channel) were perifused (flow rate = 200 μl/min), and ISR was measured in response to glucose and exposure to incrementally increasing concentrations of aqueous NaHS as indicated. Data are average ± standard error (SE), *n* = 2. (C) Cytosolic Ca^2+^ in single isolated islets were measured in response to glucose and NaHS. Representative response from four different experiments measuring responses (which were not averaged due to the use of slightly different timings of the protocols). Raw data can be found in a Source Data file named ‘Appendix 1—figure 1—source data 1’. Appendix 1—figure 1—source data 1.Effect of NaHS on insulin secretion rate and cytosolic calcium.

**Appendix 1—figure 2.**
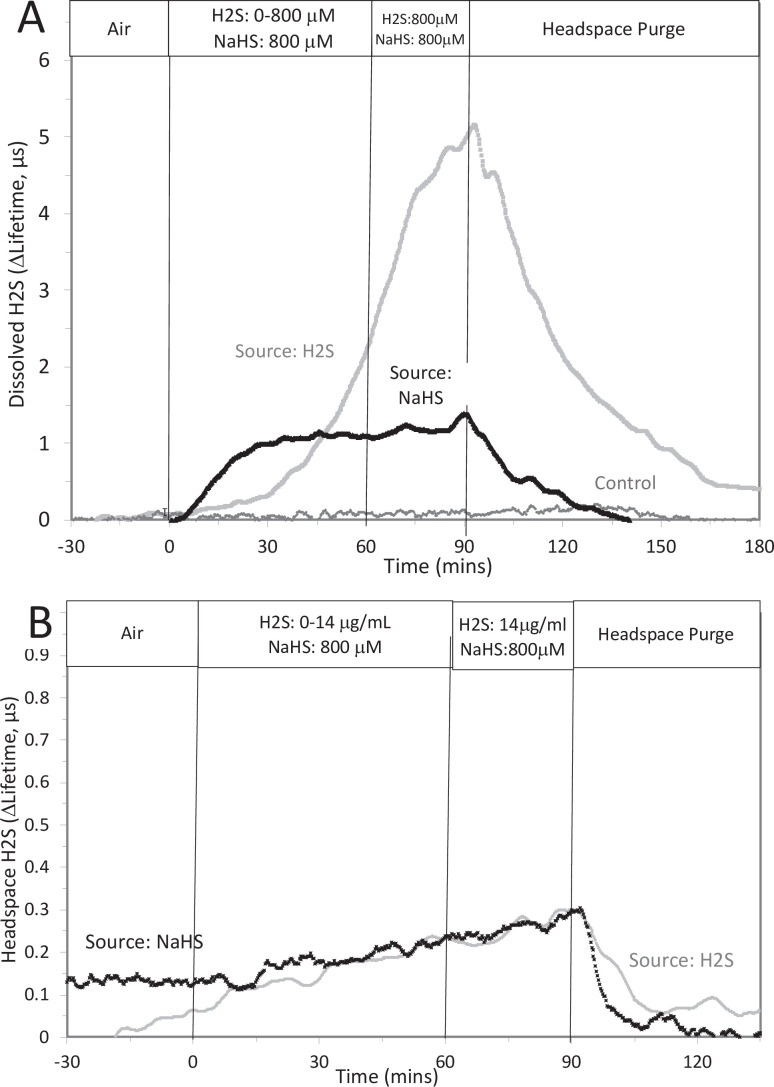
Measurement or depletion of dissolved H_2_S generated from NaHS. H_2_S was measured with sensors placed in KRB (10 ml) and the headspace of a 125 ml sealed bottle in response to either injection of 8 μM NaHS into the solution or attachment of an H_2_S-emitting permeation tube to the inlet port of the top of the bottle (rate of H_2_S permeation = 30 μg/min). Graphs represent lifetime emission reflecting H_2_S in the solution (**A**) or the headspace (**B**) from either H_2_S, NaHS, or no H_2_S (control). Legends indicate the source of the H_2_S. The concentration in solution of H_2_S from 800 μM NaHS, if equilibrium between H_2_S and HS was reached in the solution at pH 7.2 is 300 μM (calculated from [H_2_S] /([H_2_S] +[HS]) = 0.37). The concentration in solution in equilibrium with the headspace after permeation with H_2_S gas = 788 µM (calculated from Equation 1 in the main text ([H_2_S_aq_] = 14,000 ng/ml × 55.5/1000)). The maximal amount of H_2_S that could be in the headspace from NaHS in solution was calculated to be 2.5 μg/ml assuming all of the 800 μM NaHS diffused into the headspace. Raw data can be found in a Source Data file named ‘ Appendix 1—figure 2—source data 1’. Appendix 1—figure 2—source data 1.Measurement of depletion of dissolved hydrogen sulfide generated from NaHS.

**Appendix 1—figure 3.**
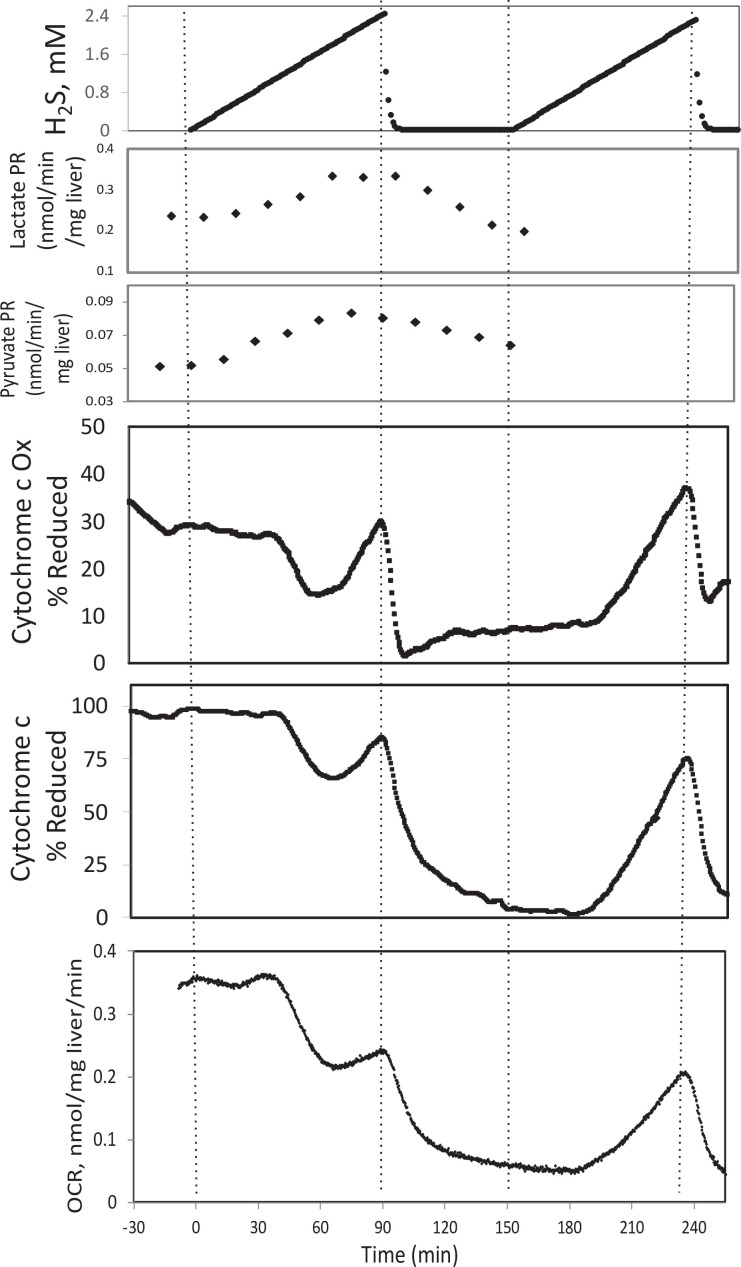
Effect of H_2_S on electron transport chain (ETC) and lactate/pyruvate in rat liver. Liver slices were exposed to increasing levels of H_2_S until the gas was purged as indicated and an additional ramp of H_2_S was implemented. O_2_ consumption rate (OCR), reduced cytochrome c, and cytochrome c oxidase were measured in real time. Fractions were collected for subsequent measurement of lactate and pyruvate. Raw data can be found in a Source Data file named ‘Appendix 1—figure 3—source data 1’. Appendix 1—figure 3—source data 1.Effect of hydrogen sulfide on metabolism in liver.

**Appendix 1—figure 4.**
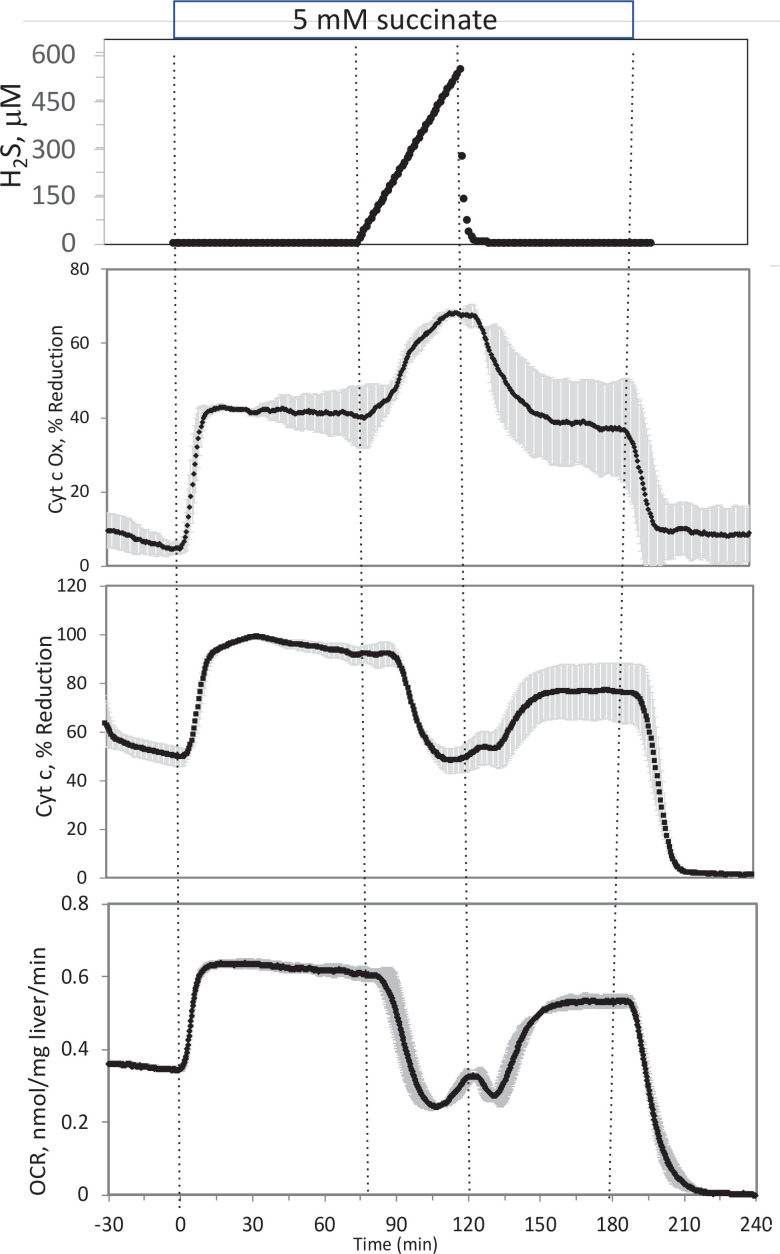
Effect of H_2_S on electron transport chain (ETC) in rat liver. In the presence of 5 mM succinate (*n* = 2), liver slices were exposed to increasing levels of H_2_S until the gas was purged as indicated. O_2_ consumption rate (OCR), reduced cytochrome c, and cytochrome c oxidase were measured in real time. Raw data can be found in a Source Data file named ‘Appendix 1—figure 4—source data 1’. Appendix 1—figure 4—source data 1.Effect of hydrogen sulfide on electron transport in liver.

## Data Availability

Data for all graphs are contained in Excel files named as the same name as the Figure followed by Source Data.

## Additional files

Transparent reporting form

## Appendix 1

### Appendix methods

#### Chemicals

Antimycin A, glucose, potassium cyanide (KCN), and succinate were purchased from Sigma-Aldrich.

#### Preparation of liver slices

Liver pieces from rats were prepared as previously described (Rountree et al., 2016). Ten pieces (about 0.25 × 1 mm [mass = 2.5–3.5 mg per piece]) were loaded into each tissue perifusion chamber without succinate, or four pieces for chambers with succinate for each analysis. The chosen size of the tissue slices was a tradeoff between avoiding hypoxia and retaining a functional unit unperturbed by the trauma of cut tissue. Although there are reports that preparation of liver slices can result in permeabilization of the cells (Kuznetsov et al., 2002), experiments comparing uptake and ^14^C-sucrose and ^3^H-H_2_O did not reveal significant membrane permeability in liver slices or retina either before or after perifusion (data not shown). At the end of each experiment, liver samples were weighed, and O_2_ consumption rate (OCR) measurements were normalized to this mass. Liver pieces had no Cytodex bead layering, but otherwise had the same loading procedure as described for retina chambers, the same supplemented KRB was continuously flowed at a rate of about 95 μl/min after initially loading the tissue at 30 μl/min. Procedures were approved by the University of Washington Institutional Animal Care and Use Committee.

#### Measurement of H_2_S in solution and headspace

H_2_S was measured indirectly by its effect on the emission of a platinum porphyrin (platinum tetrapentafluorophenyl porphyrin) dissolved and baked into a polycarbonate-silicone copolymer (Sweet et al., 2002). O_2_ shortens the lifetime of the emission energy, and H_2_S competes with O_2_’s effect. Therefore, H_2_S increases the lifetime, and at constant O_2_ (and temperature), the lifetime can be used as a measure of changes in both gaseous and dissolved H_2_S. The platinum porphyrin was affixed to the walls and bottom of 125 ml clear glass bottles, and the lifetime was measured with optical fibers as previously described (Gilbert et al., 2008).

### Appendix results

#### Effect of NaHS on glucose-stimulated insulin secretion rate by isolated rat islets

To test the ability of NaHS to emulate the of H_2_S, islets in the flow culture system at incrementally increasing concentrations of NaHS. No effect was seen at concentration below 1 μM (Appendix 1—figure 1A), a range where dissolved H_2_S had both stimulatory and inhibitory effects on insulin secretion rate (ISR). At higher concentrations, NaHS inhibited ISR where the IC_50_ was about 30 μM and each concentration change reached a new steady state within 10–15 min (Appendix 1—figure 1B). The effect of NaHS on glucose-stimulated cytosolic Ca^2+^ was measured by incrementally increasing the concentration of dissolved H_2_S in the inflow (Appendix 1—figure 1C). The response did not reach steady state in between increases in H_2_S, so the concentration dependency was not fully resolved. But in contrast to the brisk increase in cytosolic Ca^2+^ observed in response to H_2_S, at all concentrations of NaHS tested Ca^2+^ was decreased.

The question arises as to why the response to NaHS did not recapitulate the effects seen by H_2_S as would be expected if NaHS and H_2_S immediately equilibrated. However, as has been pointed out, H_2_S is very volatile and the release of dissolved H_2_S into the headspace is recognized to be an obstacle to its study (DeLeon et al., 2012). We predicted that volatility of H_2_S explains the difference between effects of H_2_S and NaHS. To test this, H_2_S was measured in 10 ml of KRB contained in a sealed 125 ml bottle, while simultaneously measuring the H_2_S in the headspace above the solution. This was done two ways: with NaHS added to the solution at a concentration of 800 μM (where at pH = 7.2 equilibrium concentration of H_2_S is 300 μM), and in another sealed bottle when H_2_S was permeated into the headspace until it reached 14 μg/ml (a concentration that results in dissolved concentration of 788 μM H_2_S from Equation 1 in the main text). Using H_2_S permeation tubes to increase the concentration of H_2_S in the headspace, the H_2_S measured (as lifetime of the dye emission) in the solution of the bottle increased, whereas the increase in response to NaHS was barely detectable (Appendix 1—figure 2A). Because the solubility of H_2_S in KRB is about a hundred times that of O_2_, the sensitivity of the lifetime of the dye emission is very high in solution. The sensitivity of the lifetime to H_2_S signal in gas was much lower and the time-dependent increase in H_2_S into the headspace was near the detection limit. Nonetheless, upon purging the bottle, the signals from H_2_S generated from both the H_2_S permeation tube and NaHS in solution clearly decreased (Appendix 1—figure 2B), confirming transfer of H_2_S from the NaHS in solution.

#### Effect of H_2_S on liver in the presence and absence of a mitochondrial fuel (succinate)

To test the ability of our system to measure the effects of H_2_S, rat liver pieces were placed into the perfusion chambers and exposed to steadily increasing concentrations of dissolved H_2_S. The first effects observed in response to changes in H_2_S were the increased production rates of lactate and pyruvate (Appendix 1—figure 3). About 30 min following the start of the ramp increase in H_2_S, OCR, reduced cytochrome c, and cytochrome c oxidase all decreased very precipitously for about 20 min, followed by a sudden increase until H_2_S was purged from the system (Appendix 1—figure 3). During the post-H_2_S phase, all three of these parameters remained low. After a second introduction of H_2_S, OCR, reduced cytochrome c, and cytochrome c oxidase all increased about 30 min following the start of the ramp. These waveforms suggest multiple points of action by H_2_S and is consistent with a scenario where H_2_S can irreversibly inhibit complex one or a step upstream from complex 1 (consistent with decreased cytochromes and OCR), but also can supply electrons directly to cytochrome c when the level of H_2_S reaches higher concentrations.

In the presence of a fuel that enters at complex 2 (succinate), OCR, reduced cytochrome c, and reduced cytochrome c oxidase all increased (Appendix 1—figure 4). Under these conditions, H_2_S decreased OCR and cytochrome c reduction, but increased cytochrome c oxidase reduction, suggesting that H_2_S was inhibiting both at complexes 1 and 4. In contrast to the experiments done in the absence of succinate, post-H_2_S metabolism was only slightly different than pre-H_2_S energy state reflecting the continued supply of electrons to the ETC when complex one is still inhibited, while complex four inhibition by H_2_S seemed to be reversible. Following washout of both H_2_S and succinate, reducing power in the mitochondria fell to near 0, as would be expected in the absence of complex one activity.

### Appendix discussion

#### Contrasting effect of H_2_S and NaHS on ISR and Ca^2+^

The assumption made in most studies of H_2_S, is that NaHS or Na_2_S would rapidly equilibrate with the protonated form of the acid. Whether NaHS or H_2_S is added to the solution, the same amount of H_2_S would be present in solution after a short equilibration time. Our studies showed that NaHS does not recapitulate the effects of H_2_S and that this is caused by rapid depletion of H_2_S from the solution via transference to the headspace. The amount of dissolved H_2_S in a ml of aqueous solution is about 0.35% of that in equilibria with 1 ml of headspace. This means that unless the volume of the headspace is on the order of 1/100th of the volume of the solution, then the headspace will act as an infinite sink for H_2_S in the solution and NaHS will not result in a significant increase in dissolved H_2_S. This is the case in static or perifusion systems that are commonly used to perform ISR assays. The data support the guidance that when investigating the physiological effects of H_2_S in equilibrium with HS^−^, H_2_S should be supplied via the headspace and calls into question the use of H_2_S donor molecules where the effects of HS^−^ but not H_2_S will be observed.

Having shown that H_2_S in solution generated from NaHS is depleted as a result of transfer into the headspace, we considered two scenarios to interpret the effects of NaHS. Either protonation of HS^−^ is rapid relative to the diffusion of dissolved H_2_S into the headspace and HS^−^ and H_2_S reach equilibrium. Alternatively, diffusion of dissolved H_2_S into the headspace is fast, and HS^−^ generated from NaHS exists in solution in the presence of very low levels of H_2_S. If the equilibrium between HS^−^ and H_2_S was reached however, then the effect of NaHS would be the same as the effect of H_2_S, except that the concentration dependency would be right-shifted relative to when H_2_S from the headspace is the source of sulfide. However, no concentration of NaHS resulted in an increase of either ISR or Ca^2+^. Thus, it appears that consistent with the low solubility of gases in aqueous solution, and the relatively slow rate of protonation and deprotonation for weak acids, NaHS generates only HS^−^ when the headspace is similar or larger than the solution, and that HS^−^ is inhibitory for both ISR and Ca^2+^.

#### Control and effects of H_2_S on liver

To illustrate its use, we exposed liver to H_2_S, an established cell signal with multiple effects and mechanisms of action. Both reported mechanisms of action of H_2_S in the ETC were observed in this study: inhibition of cytochrome c oxidase (Khan et al., 1990) and direct transfer of electrons to cytochrome c (Vitvitsky et al., 2018).

As the concentration of dissolved H_2_S increased, reduced cytochrome c, reduced cytochrome c oxidase, and OCR all decreased reflecting indicating inhibition of step(s) upstream of complex 1 (which appeared irreversible), and subsequently all three parameters increased consistent with donation of electrons directly from H_2_S. In the presence of a mitochondrial fuel entering at complex 2 (succinate), H_2_S caused a decrease in reduced cytochrome c and OCR, while increasing reduced cytochrome c oxidase consistent with the simultaneous inhibition of complexes 1 and 4. This analysis highlights the benefit of measuring reductive states of cytochromes concomitantly with OCR: interpreting observed changes in OCR in terms of the mechanisms mediating the changes is not possible with OCR measurements alone. The factors mediating the changes in OCR are distinguished by concomitant measurement of both electron pool sizes in the ETC (cytochromes) and flux of electrons (OCR). Overall, the method was able to characterize complex and multiple effects of H_2_S on the ETC.
