# Correction: Fluidics system for resolving concentration-dependent effects of dissolved gases on tissue metabolism

**DOI:** 10.7554/eLife.77538

**Published:** 2022-02-14

**Authors:** Varun Kamat, Brian M Robbings, Seung-Ryoung Jung, John Kelly, James B Hurley, Kenneth P Bube, Ian R Sweet

**Keywords:** Mouse, Rat

Kamat V, Robbings BM, Jung SR, Kelly J, Hurley JB, Bube KP, Sweet IR. Fluidics system for resolving concentration-dependent effects of dissolved gases on tissue metabolism. *eLife* **10**:e66716. doi: 10.7554/eLife.66716

Published online Nov 4 2021

In the original publication, we used an incorrect formulation of Henry’s law resulting in the underestimation of the dissolved concentration of H_2_S that was in equilibrium with the headspace. Since the paper is a methods paper that was published as a Tools and Resources article, there are no implications regarding the operation of the instrument. However, the effects of H_2_S on islet and liver metabolism and function occurred at higher concentrations than reported, and in the corrected manuscript we have now rectified this discrepancy. The major changes to the paper are in the x axes of Figure 6 and the Figures in the Appendix, and in text that involved the concentration range of effects.

**#1 (Introduction)**

Corrected Text:

In addition, the study of dissolved gases is hampered by the volatility of dissolved gases under conditions where the headspace is not supplied with equilibrium levels of the gas (DeLeon et al., 2012).

Original Text:

In addition, the study of trace gases is hampered by the volatility of dissolved gases under conditions where the headspace is not supplied with equilibrium levels of the gas (DeLeon et al., 2012).

**#2 (Results)**

Corrected Text:

When H_2_S was increased until a steady state of 240 μM was reached, ISR from pancreatic islets increased by 35% relative to ISR at 20 mM glucose (Figure 6A). The increased ISR was sustained for 3 hr. The effect of H_2_S was reversible. After purging it from the system, ISR rapidly returned to levels that occurred prior to H_2_S exposure. In the presence of 3 mM glucose, H_2_S had no effect on ISR (data not shown), supporting the idea that this reflects a physiologic response of ISR to H_2_S. To demonstrate the ability of the flow system to more fully characterize the time- and concentration dependency of ISR on H_2_S, we measured ISR at steady-state concentrations of H_2_S from 80 to 780 μM. Notably, between 180 and 780 μM H_2_S (Figure 6B), the initial period of stimulation of ISR (peaking between 1 and 1.5 hr after the start of the ramp of H_2_S) was insensitive to the concentration of H_2_S.

Original Text:

When H_2_S was increased until a steady state of 0.44 μM was reached, ISR from pancreatic islets increased by 35% relative to ISR at 20 mM glucose (Figure 6A). The increased ISR was sustained for 3 hours. The effect of H_2_S was reversible. After purging it from the system, ISR rapidly returned to levels that occurred prior to H_2_S exposure. In the presence of 3 mM glucose, H_2_S had no effect on ISR (data not shown), supporting the idea that this reflects a physiologic response of ISR to H_2_S. To demonstrate the ability of the flow system to more fully characterize the time- and concentration dependency of ISR on H_2_S, we measured ISR at steady-state concentrations of H_2_S from 0.15 to 1.42 μM. Notably, between 0.33 and 1.42 μM H_2_S (Figure 6B), the initial period of stimulation of ISR (peaking between 1 and 1.5 hr after the start of the ramp of H_2_S) was insensitive to the concentration of H_2_S.

**#3 (Results)**

Corrected Text:

The H_2_S in solution emanating from the headspace was higher than that achieved from NaHS, but notably it declined when the headspace was purged. Although the amount in the headspace was close to the detection limit of the H_2_S measurement method, it was clear from the sharp decline in H_2_S after unsealing the bottle, that H_2_S from the NaHS had indeed transferred into the headspace. Thus, these data support a scenario where differences in effects of H_2_S and NaHS occur due to the absence of H_2_S in solution containing NaHS and HS^−^ is inhibitory for both ISR and Ca^2+^.

Original Text:

The H_2_S in solution emanating from the headspace was many-fold higher than that achieved from NaHS. Although the amount in the headspace was close to the detection limit of the H_2_S measurement method, it was clear from the sharp decline in H_2_S after unsealing the bottle, that H_2_S from the NaHS had indeed transferred into the headspace. Thus, these data support a scenario where differences in effects of H_2_S and NaHS occur due to the absence of H_2_S in solution containing NaHS and that HS^−^ is inhibitory for both ISR and Ca^2+^.

**#4 (Results)**

Corrected Text:

In the presence of a TCA cycle intermediate (succinate), H_2_S (200–300 μM) increased the reductive state of cytochrome c oxidase while decreasing OCR. This is consistent with inhibition of cytochrome c oxidase (Khan et al., 1990; Appendix 1—figure 4), which occurred at concentration similar to typical estimates of plasma concentration which range from 30 to 300 μM (Olson, 2009).

Original Text:

In the presence of a TCA cycle intermediate (succinate), H_2_S (2–3 μM) increased the reductive state of cytochrome c oxidase while decreasing OCR. This is consistent with inhibition of cytochrome c oxidase (Khan et al., 1990; Appendix 1—figure 4), which notably occurred at concentration many times lower than typical estimates of plasma concentration which range from 30 to 300 μM (Olson, 2009).

**#5 (Discussion)**

Corrected Text:

We achieved this by incorporating a unique gas equilibration system that controls abundant (blood) gases including O_2_, CO_2_, and N_2_, and by using permeation tubes to introduce and control endogenously-produced gases such as H_2_S, NO, and CO. In this report, we demonstrated the utility of this system using both a blood (O_2_) and a signaling gas (H_2_S).

Original Text:

We achieved this by incorporating a unique gas equilibration system that controls abundant (blood) gases including O_2_, CO_2_, and N_2_, and by using permeation tubes to introduce and control trace gases such as H_2_S, NO, and CO. In this report we demonstrated the utility of this system using both an abundant (O_2_) and a trace gas (H_2_S).

**#6 (Discussion)**

Corrected Text:

Gas signaling molecules (CO, NO, and H_2_S) are generated in most tissues and have wide-ranging effects on function,

Original Text:

Trace gas signaling molecules (CO, NO, and H_2_S) are generated in most tissues and have wide-ranging effects on function,

**#7 (Discussion)**

Corrected Text:

Our system bore out this prediction revealing stimulatory effects of H_2_S on ISR that had not been apparent when exposing islets to a donor of H_2_S (NaHS). H_2_S at concentrations (between 140 and 280 μM) enhanced glucose-stimulated ISR, which remained elevated for at least 4 hr.

Original Text:

Our system bore out this prediction revealing stimulatory effects of H_2_S on ISR that had not been apparent when exposing islets to a donor of H_2_S (NaHS). H_2_S at low concentrations (between 0.25 and 0.5 μM) enhanced glucose-stimulated ISR, which remained elevated for at least 4 hr.

**#8 (Discussion)**

Corrected Text:

It is notable that the range of concentrations that induced changes in ISR, and above which caused inhibition of OCR in liver are in the range of typical estimates of plasma concentration which range from 30 to 300 μM (Olson, 2009). Therefore, it suggests that intracellular effects of H_2_S could be mediated by H_2_S derived from the blood as well as H_2_S endogenously produced by cells.

Original Text:

It is notable that the range of concentrations that induced changes in ISR, and above which caused inhibition of OCR in liver are many times lower than typical estimates of plasma concentration which range from 30 to 300 μM (Olson, 2009). Although it is possible that in vivo, tissue is much less sensitive to H_2_S than in vitro, it seems more likely that assays that measure plasma H_2_S result in a significant overestimate coming from related sulfur-containing compounds.

**#9 (Methods)**

Corrected Text:

Henry’s constant is defined as

$$Hc=1000\times [gas{]}_{aq}/[gas{]}_{g}$$

where [gas]_aq_ is in μM, and [gas]_g_ is in ng/ml.

At 37 degrees dissolved O_2_ is 217 nmol/mL in KRB, which is in equilibrium with 0.3008 mg/mL of O2 in air (21%). Therefore,

$$Hc(O_{2})=1000\times [O_{2}{]}_{aq}/[O_{2}{]}_{g}=217\mu M/300,800ng/mL\times 1000=0.721$$

In order to estimate Henry’s constants for H_2_S at 37 degrees in KRB, we used measurement of solubility (reported by National Institute of Standards and Technology) normalized relative to O_2_ in the relationship

$$Hc(H_{2}S)=Solubility(H_{2}S)/Solubility(O2)\times Hc(O_{2})=0.1/0.0013\times 0.721=55.5$$

Thus, the equation relating the dissolved H_2_S concentrations to head space concentration is

(1) $$[H_{2}S{]}_{aq}=55.5\times [H_{2}S]g/1000$$

where [H_2_S_aq_] is in μM, and [H_2_S_g_] is in ng/mL

Original Text:

The amount of dissolved H_2_S in the buffer was then calculated based on the solubility of H_2_S in buffer based on Henry’s Law:

(1) $$[H_{2}S_{aq}]=[H_{2}S_{g}]\times Hc/1000$$

where Henry’s constant Hc is in 0.1 atm/M, [H_2_S_aq_] is in μM, and [H_2_S_g_] is in ng/mL

**#10 (Figure Legends)**

Corrected Text:

**Figure 6. Effect of H_2_S on insulin secretion rate (ISR) by islets**. (**A**) Rat islets (50/channel) were perifused (flow rate = 200 μL/min), and ISR was measured in response to glucose and exposure to dissolved H_2_S in the concentrations shown (data are average ± standard error [SE], n = 3 [H_2_S], n = 2 [no H_2_S], *P* < 0.05 as indicated). (**B**) ISR was measured at the indicated concentrations of dissolved H_2_S. Each curve is a single experiment. (**C**) Data from perifusions as shown in B were plotted as a function of the ISR at the peak between 1 and 1.5 hr, and the average ISR between 3 and 4 hr. (**D**) Response of cytosolic Ca^2+^ to changes in glucose concentration and exposure to 166 μM H_2_S and its washout.

Original Text:

**Figure 6. Effect of H_2_S on insulin secretion rate (ISR) by islets**. (**A**) Rat islets (50/channel) were perifused (flow rate = 200 μL/min), and ISR was measured in response to glucose and exposure to dissolved H_2_S in the concentrations shown (data are average ± standard error [SE], n = 3 [H_2_S], n = 2 [no H_2_S], *P* < 0.05 as indicated). (**B**) ISR was measured at the indicated concentrations of dissolved H_2_S. Each curve is a single experiment. (**C**) Data from perifusions as shown in B were plotted as a function of the ISR at the peak between 1 and 1.5 hr, and the average ISR between 3 and 4 hr. (**D**) Response of cytosolic Ca^2+^ to changes in glucose concentration and exposure to 0.3 μM H_2_S and its washout.

**#11 (Figure 6)**

Corrected Figure:

**Figure 1:**
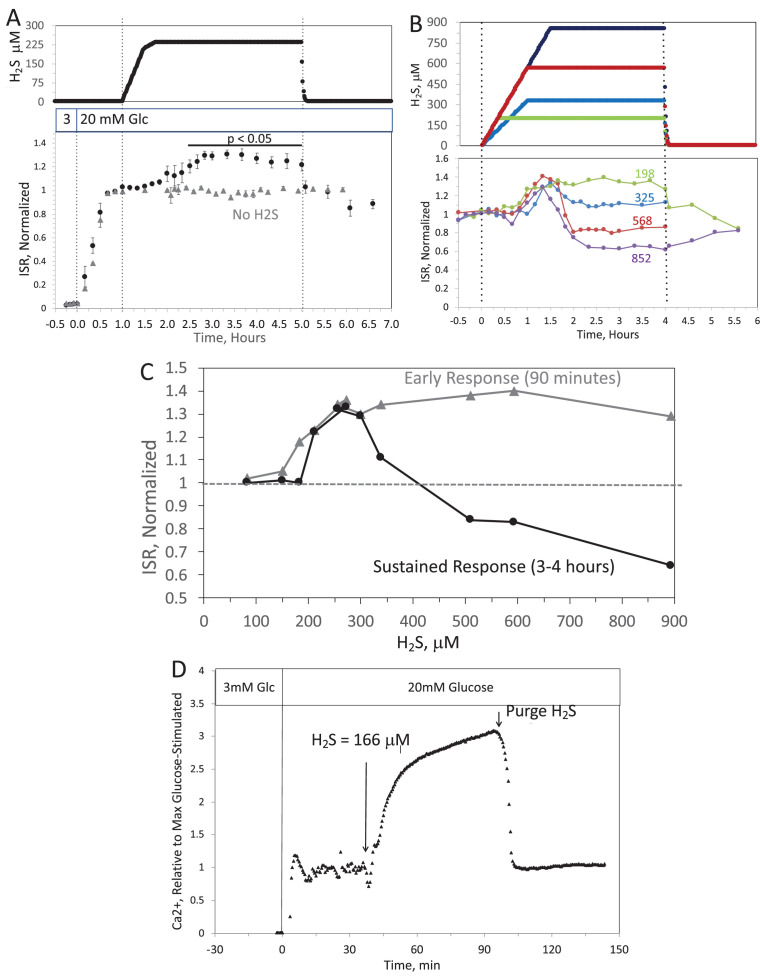


Original Figure:

**Figure 2:**
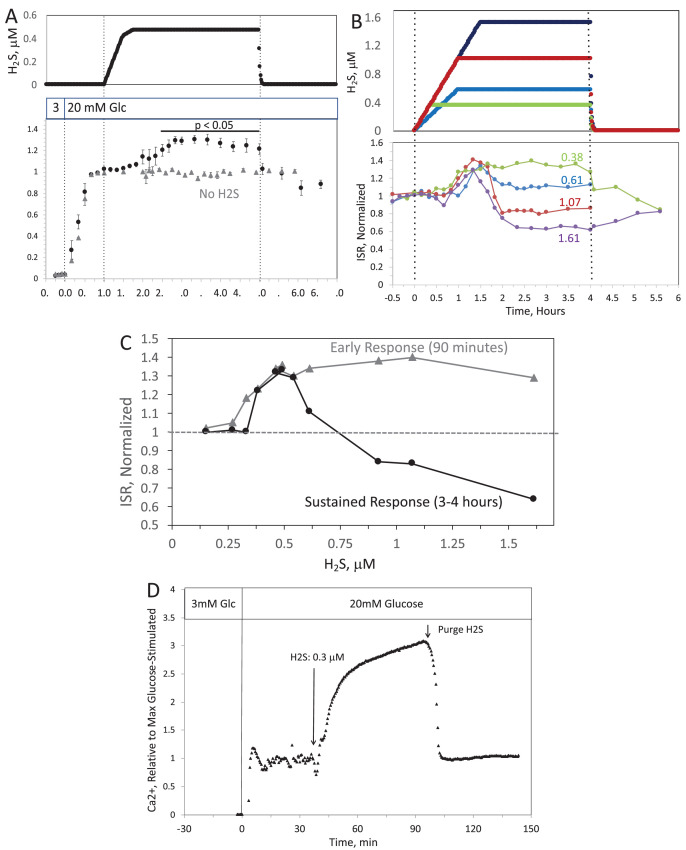


**Appendix 1**

**#12 (Appendix results)**

Corrected Text:

The response did not reach steady state in between increases in H_2_S, so the concentration dependency was not fully resolved. But in contrast to the brisk increase in cytosolic Ca^2+^ observed in response to H_2_S, at all concentrations of NaHS tested Ca^2+^ was decreased.

Original Text:

The response did not reach steady state in between increases in H_2_S so the concentration dependency was not resolved, but in contrast to the brisk increase in cytosolic Ca^2+^ observed in response to H_2_S, even at low concentrations of NaHS tested Ca^2+^ was decreased.

**#13 (Appendix results)**

Corrected Text:

This was done two ways: with NaHS added to the solution at a concentration of 800 μM (where at pH = 7.2 equilibrium concentration of H_2_S is 300 μM), and in another sealed bottle when H_2_S was permeated into the headspace until it reached 14 μg/mL (a concentration that results in dissolved concentration of 788 μM H_2_S from Equation 1 in the main text).

Original Text:

This was done two ways: with NaHS added to the solution at a concentration of 8 μM (where at pH = 7.2 equilibrium concentration of H_2_S is 3 μM), and in another sealed bottle when H_2_S was permeated into the headspace until it reached 14 μg/mL (a concentration that results in dissolved concentration of 1.4 μM H_2_S from Equation 1 in the main text).

**#14 (Appendix 1—figure 2 Legend)**

Corrected Text:

The concentration in solution of H_2_S from 800 μM NaHS, if equilibrium between H_2_S and HS was reached in the solution at pH 7.2 is 300 μM (calculated from [H_2_S] /([H_2_S] +[HS]) = 0.37). The concentration in solution in equilibrium with the headspace after permeation with H_2_S gas = 788 µM (calculated from Equation 1 in the main text ([H_2_S_aq_] = 14,000 ng/mL × 55.5/1000)). The maximal amount of H_2_S that could be in the headspace from NaHS in solution was calculated to be 2.5 μg/mL assuming all of the 800 μM NaHS diffused into the headspace.

Original Text:

The concentration in solution of H_2_S from 8 μM NaHS, if equilibrium between H_2_S and HS was reached in the solution at pH 7.2 is 3 μM (calculated from [H_2_S] /([H_2_S] +[HS]) = 0.37). The concentration in solution in equilibrium with the headspace after permeation with H_2_S gas = 1.4 µM (calculated from Equation 1 in the main text ([H_2_S_aq_] = 14,000 ng/mL × 0.1/1000)). The maximal amount of H_2_S that could be in the headspace from NaHS in solution was calculated to be 25 ng/mL assuming all of the 8 μM NaHS diffused into the headspace.

**#15 (Appendix 1—figure 1)**

Corrected Figure:

**Figure 3:**
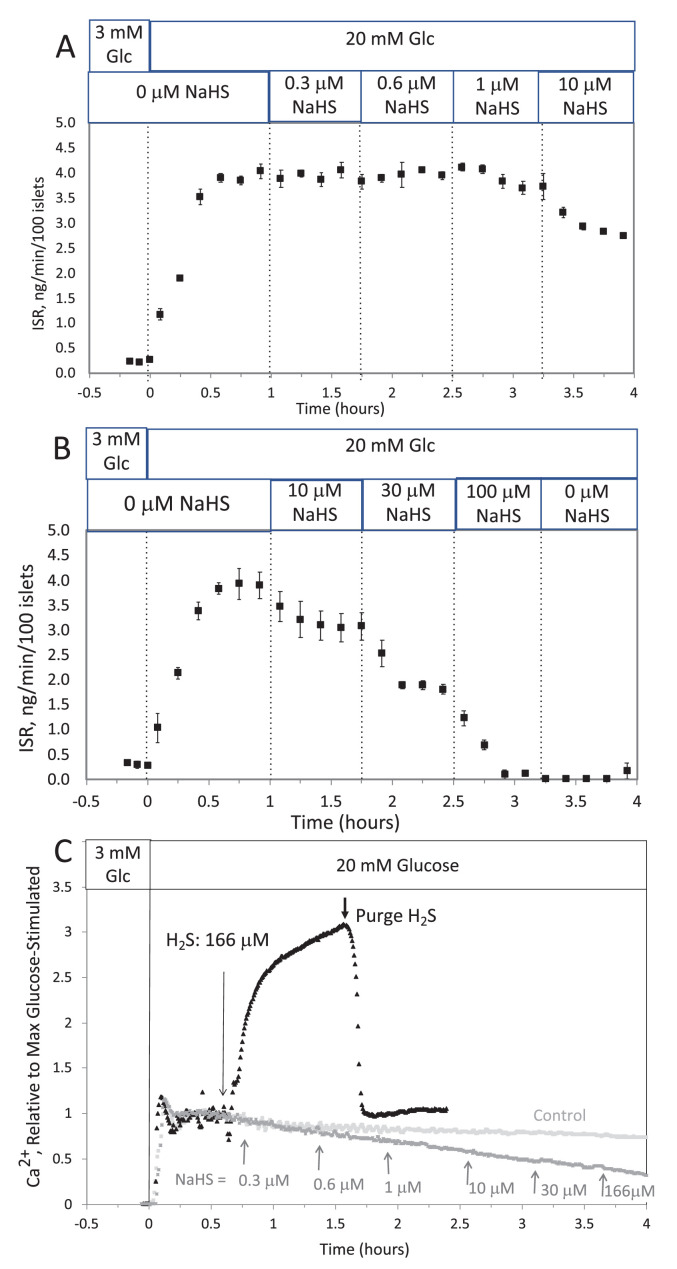


Original Figure:

**Figure 4:**
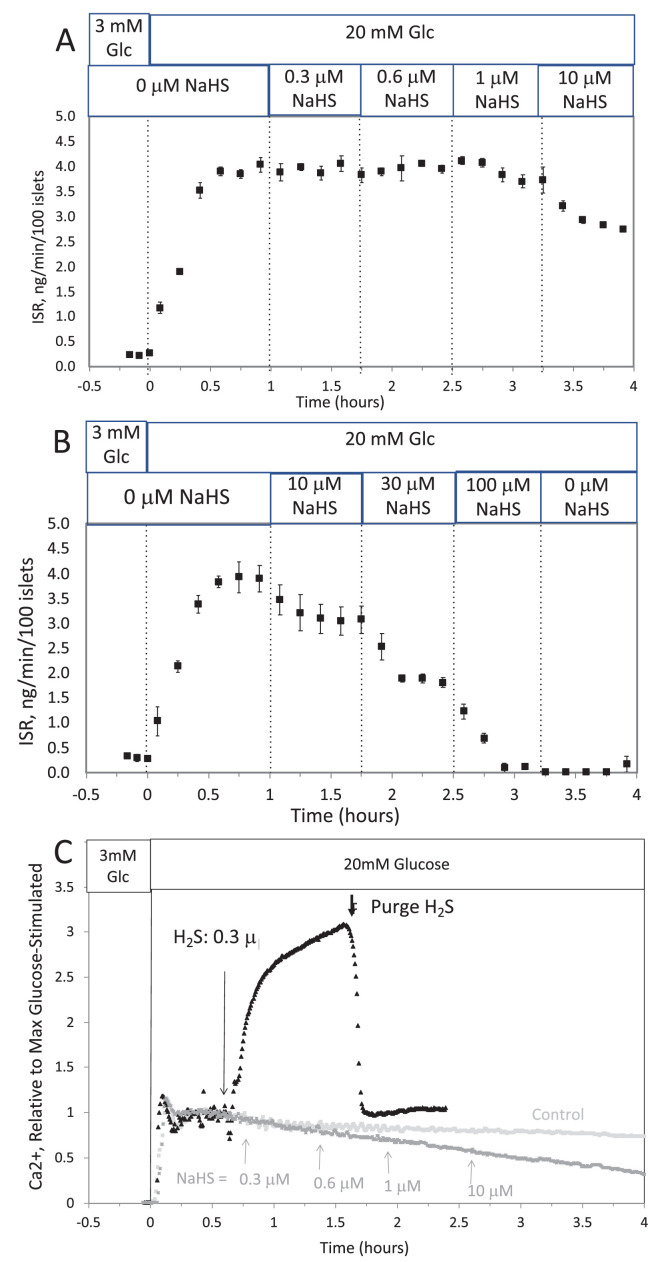


**#16 (Appendix 1—figure 2)**

Corrected Figure:

**Figure 5:**
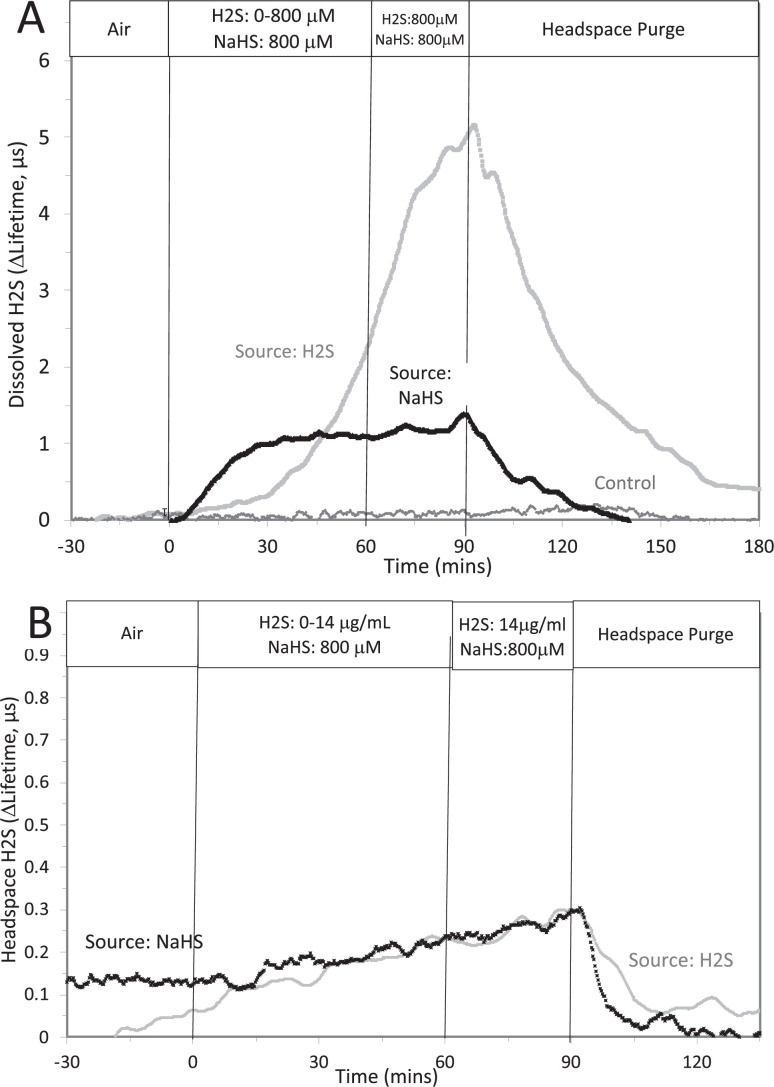


Original Figure:

**Figure 6:**
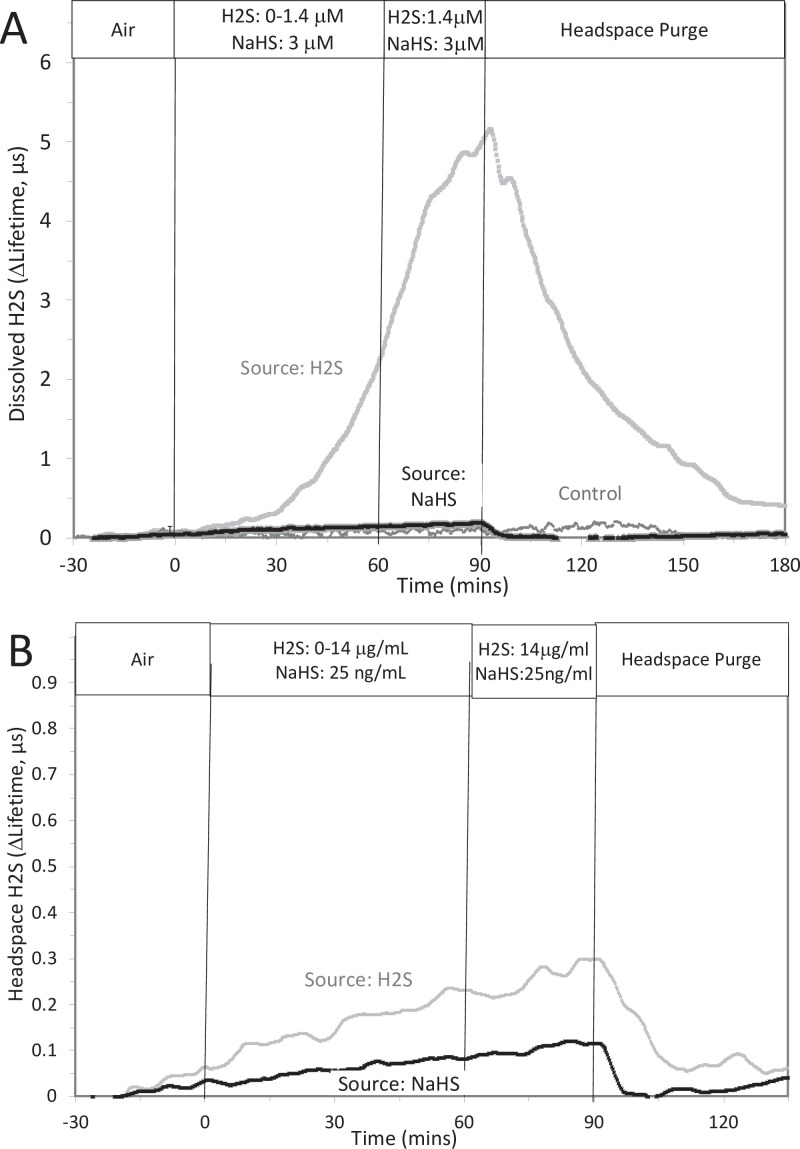


**#17 (Appendix 1—figure 3)**

Corrected Figure:

**Figure 7:**
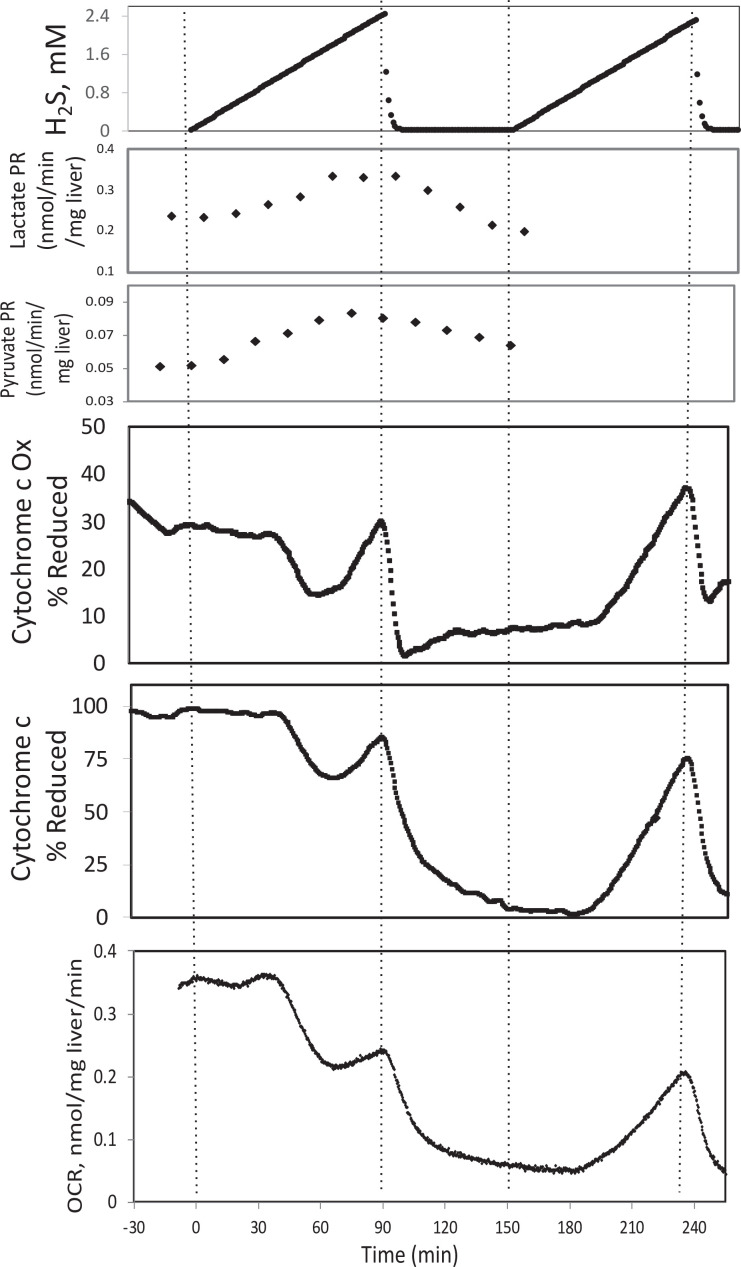


Original Figure:

**Figure 8:**
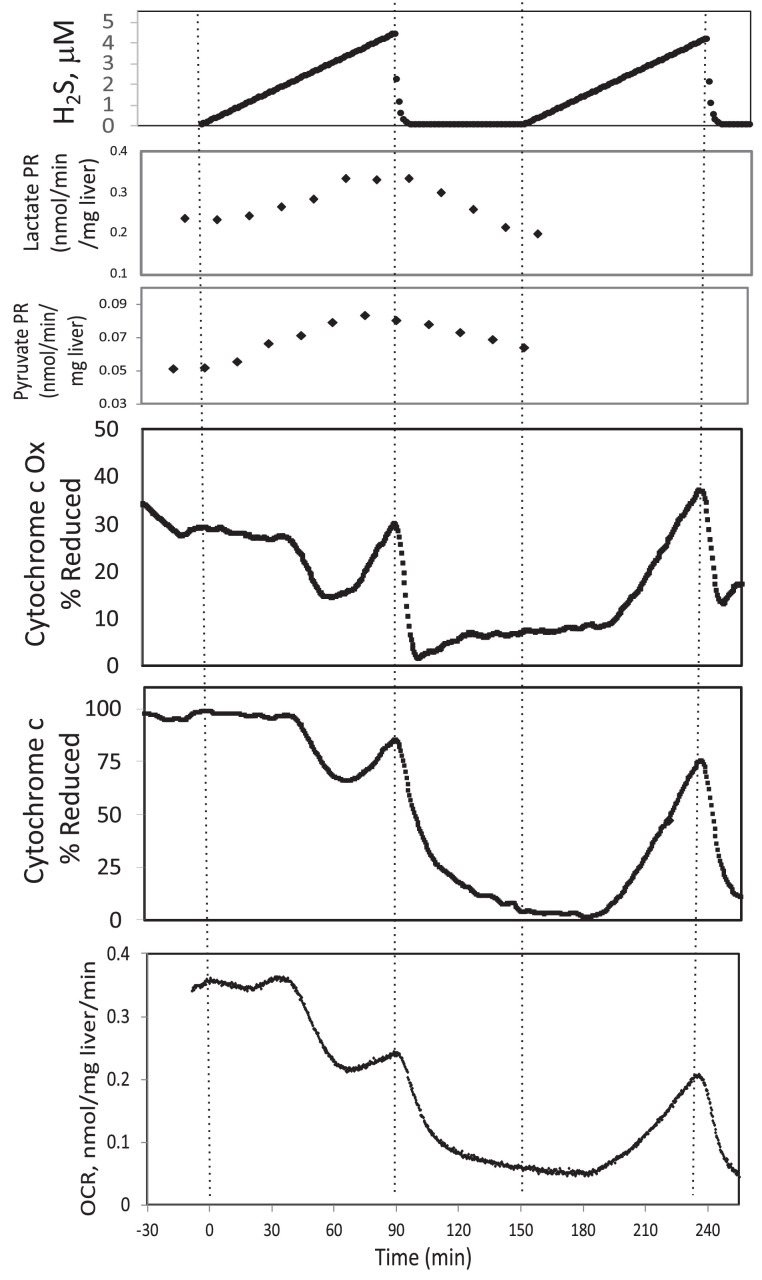


**#18 (Appendix 1—figure 4)**

Corrected Figure:

**Figure 9:**
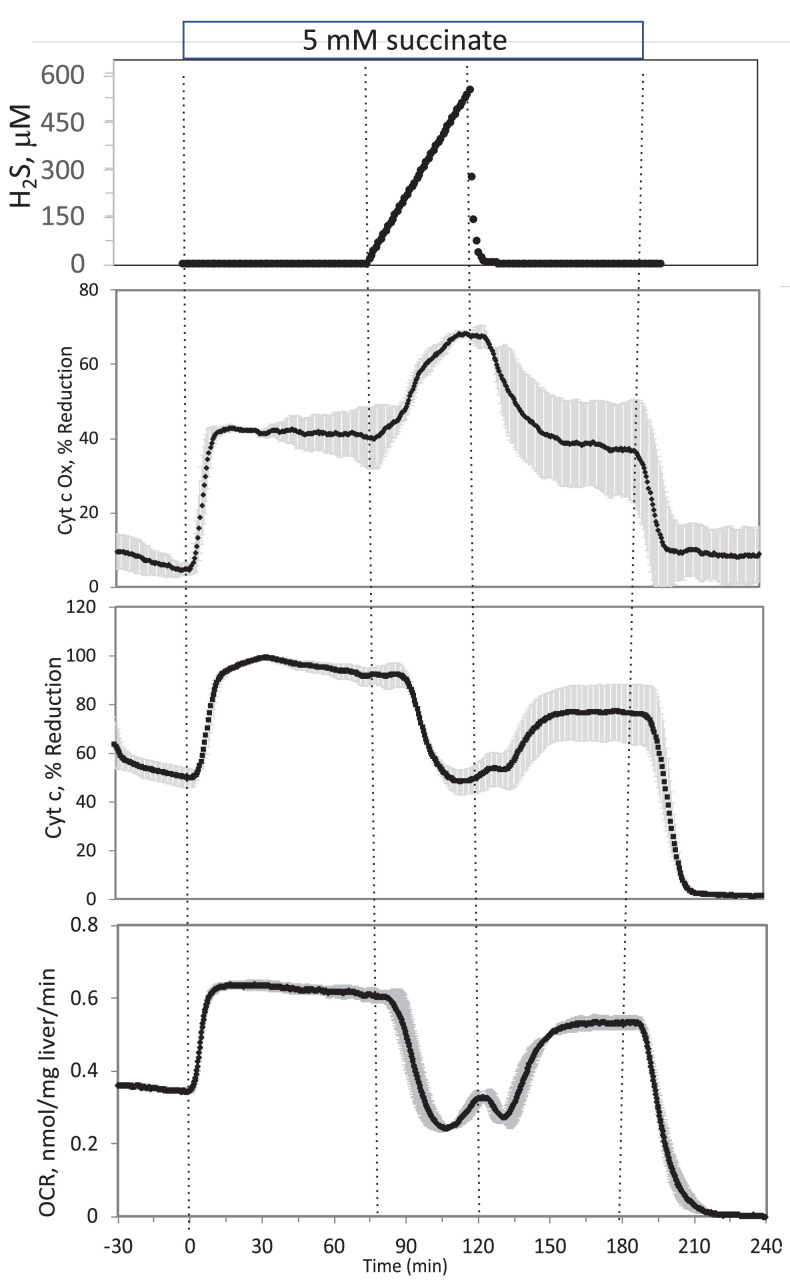


Original Figure:

**Figure 10:**
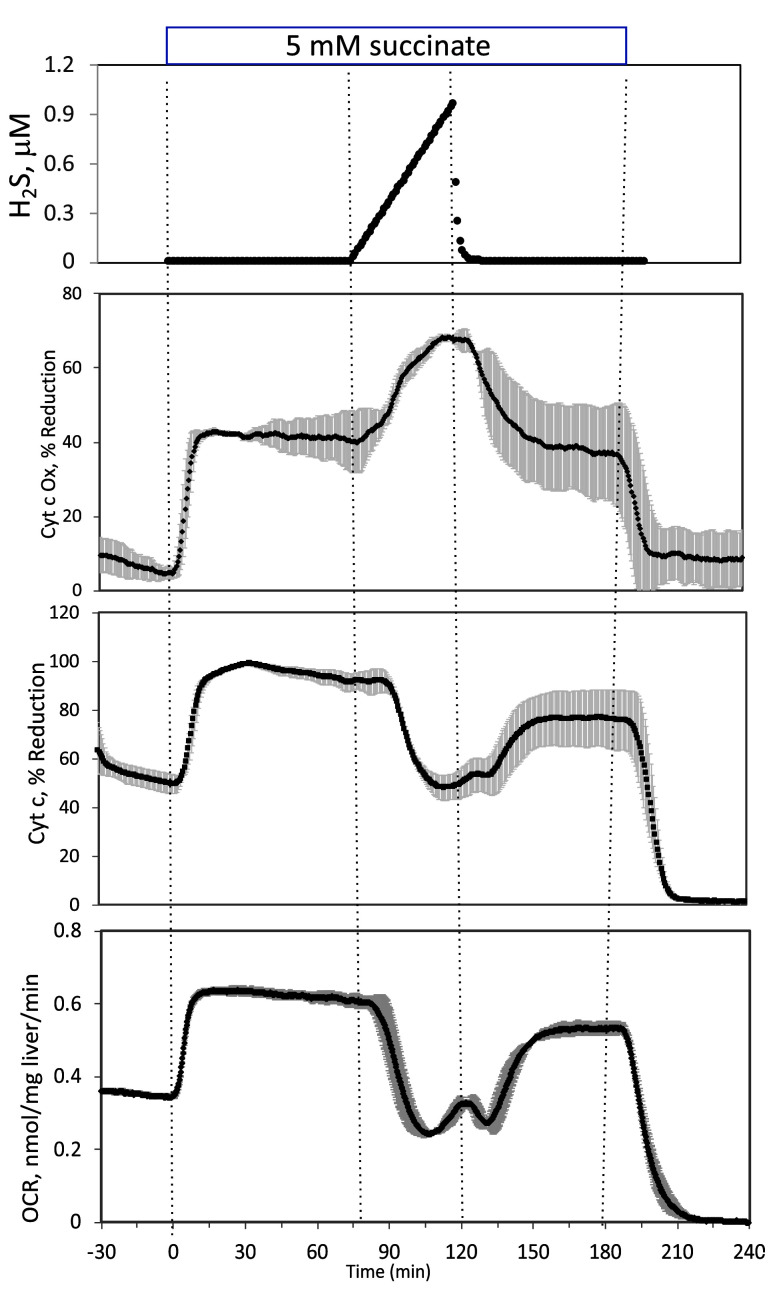


Where H_2_S had to be recalculated, the source files were corrected. These corrected sources files include:

Figure 6—source data 1

Appendix 1—Figure 2—source data 1

Appendix 1—Figure 3—source data 1

Appendix 1—Figure 4—source data 1

